# Phytochemical Diversity in Rhizomes of Three *Reynoutria* Species and their Antioxidant Activity Correlations Elucidated by LC-ESI-MS/MS Analysis

**DOI:** 10.3390/molecules24061136

**Published:** 2019-03-21

**Authors:** Izabela Nawrot-Hadzik, Sylwester Ślusarczyk, Sebastian Granica, Jakub Hadzik, Adam Matkowski

**Affiliations:** 1 Department of Pharmaceutical Biology and Botany, Wroclaw Medical University, 50-367 Wrocław, Poland; izabela.nawrot-hadzik@umed.wroc.pl (I.N.-H.); sylwester.slusarczyk@umed.wroc.pl (S.Ś.); 2 Department of Pharmacognosy and Molecular Foundations of Phytotherapy, Warsaw Medical University, 02-097 Warszawa, Poland; sgranica@wum.edu.pl; 3 Department of Dental Surgery, Wroclaw Medical University, 50-425 Wrocław, Poland; jakub.hadzik@umed.wroc.pl; 4 Botanical Garden of Medicinal Plants, Wroclaw Medical University, 50-367 Wrocław, Poland

**Keywords:** proanthocyanidins, *Polygoni cuspidati rhizoma*, mass spectrometry, stilbenoids

## Abstract

The rhizome of *Reynoutria japonica* is a well-known traditional herb (Hu zhang) used in East Asia to treat various inflammatory diseases, infections, skin diseases, scald, and hyperlipidemia. It is also one of the richest natural sources of resveratrol. Although, it has been recently included in the European Pharmacopoeia, in Europe it is still an untapped resource. Some of the therapeutic effects are likely to be influenced by its antioxidant properties and this in turn is frequently associated with a high stilbene content. However, compounds other than stilbenes may add to the total antioxidant capacity. Hence, the aim of this research was to examine rhizomes of *R. japonica* and the less studied but morphologically similar species, *R. sachalinensis* and *R.* x *bohemica* for their phytochemical composition and antioxidant activity and to clarify the relationship between the antioxidant activity and the components by statistical methods. HPLC/UV/ESI-MS studies of three *Reynoutria* species revealed 171 compounds, comprising stilbenes, carbohydrates, procyanidins, flavan-3-ols, anthraquinones, phenylpropanoids, lignin oligomers, hydroxycinnamic acids, naphthalenes and their derivatives. Our studies confirmed the presence of procyanidins with high degree of polymerization, up to decamers, in the rhizomes of *R. japonica* and provides new data on the presence of these compounds in other *Reynoutria* species. A procyanidin trimer digallate was described for the first time in, the studied plants. Moreover, we tentatively identified dianthrone glycosides new for these species and previously unrecorded phenylpropanoid disaccharide esters and hydroxycinnamic acid derivatives. Furthermore, compounds tentatively annotated as lignin oligomers were observed for the first time in the studied species. The rhizomes of all *Reynoutria* species exhibited strong antioxidant activity. Statistical analysis demonstrated that proanthocyanidins should be considered as important contributors to the total antioxidant capacity.

## 1. Introduction

In East Asia, the rhizome of *Reynoutria japonica* Houtt. (syn. *Fallopia japonica* [Houtt.] Ronse Decr., obsolete syn. *Polygonum cuspidatum* Sieb. & Zucc.) is a well-known traditional herb (Hu zhang, Polygoni cuspidatae rhizoma) used to treat various inflammatory diseases, infections, skin diseases, scald, hyperlipidemia, etc. [1]. It is also one of the richest natural source of resveratrol (free and glycosylated) which has proven antioxidant activity [2]. In Europe, *R. japonica* has until recently been considered primarily as a troublesome invasive species that threatens native vegetation. However, rhizome of *R. japonica* (*Polygoni cuspidati rhizoma*) has been recently included in the European Pharmacopoeia [3] along with many other traditional Chinese herbs. A morphologically similar species from this genus—*R. sachalinensis* (F. Schmidt) Nakai, (syn. *F. sachalinensis* (F. Schmidt) Ronse Decr., *P. sachalinense* F. Schmidt) and a hybrid between them, *R.* x *bohemica* Chrtek & Chrtková (syn. *F*. x *bohemica* (Chrtek & Chrtková) J.P. Bailey) are not considered as equivalent medicinal plants. Both are also noxious invasive weeds outside their native distribution areas, However, *R. sachalinensis* has been to some extent used traditionally as a herbal medicine in Japan and China for treatment of arthralgia, jaundice, amenorrhea, coughs, scalds and burns, traumatic injuries, carbuncles and sores [4]. Earlier studies revealed striking differences in the metabolic profiles of these three species. *R. sachalinensis rhizomes* contained lower quantities of anthraquinones than rhizomes of *R. japonica* and significantly higher amounts of phenylpropanoid-derived disaccharide esters. Moreover, no stilbenes were detected *in R. sachalinensis*, in contrast to the rich presence of these compounds in rhizomes of *R. japonica*. The phytochemical profile of *R.* x *bohemica* was intermediate between the two parent species [5]. Some of the therapeutic effects of the studied species are likely to be influenced by their antioxidant properties and this in turn is frequently associated with a high stilbene content [6]. However, some researchers showed no correlation between the content of resveratrol or emodin and antioxidant activity in the obtained extracts and fractions from *R. japonica* [7]. Ding et al. [8] revealed a high positive correlation value for flavanol gallate, resveratrol and catechin but low for piceid, questin (or physcion), and no correlation to emodin-8-*O*-glucoside, questin (or physcion) and emodin. Moreover, in a study by Pan et al. [9], an ethanol extract of *R. japonica* had a stronger antioxidant activity than resveratrol. These data suggested that compounds other than stilbenes may contribute to the total antioxidant capacity. It is thus worth looking more closely at the phytochemical profile of rhizomes from all three *Reynoutria* species.

The aim of the present study was to examine rhizomes of the invasive *Reynoutria* species from the wild population in Poland for their phytochemical composition and antioxidant activity. To clarify the relationship between the antioxidant activity and compounds present in the extracts and fractions, the statistical analysis was performed involved the LC-MS data and results from antioxidant assays. 

## 2. Results and Discussion

### 2.1. Mass Spectra Analysis, Annotation and Identification of Major Constituents in Extracts and Fractions

**Figure 1:**
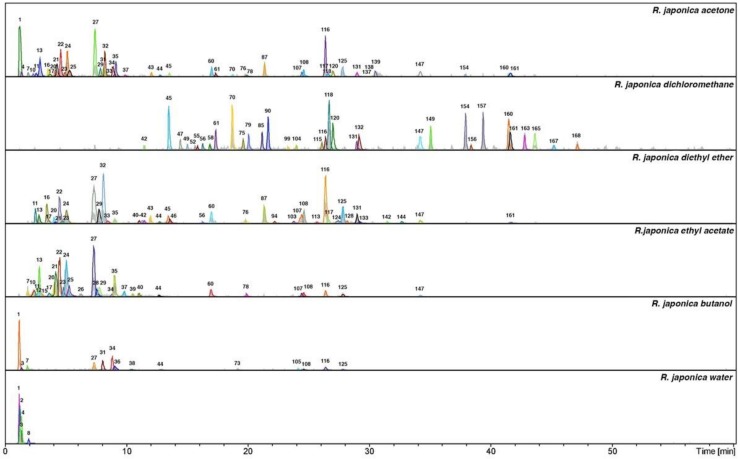
Total ion chromatograms in negative ionization mode and dissect chromatograms of *Reynoutria japonica* extract and fractions. Deconvolution of an LC/MS mass chromatogram was carried out by using the Bruker’s Dissect algorithm. Peak numbers are explained in Table 1.

**Figure 2:**
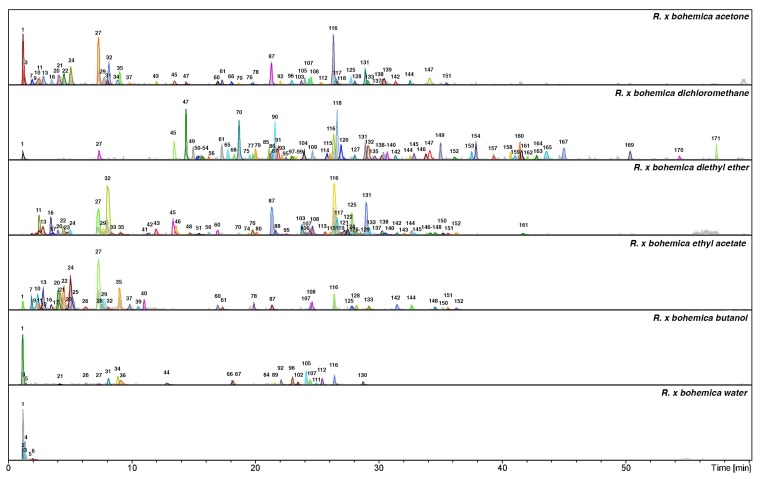
Total ion chromatograms in negative ionization mode and dissect chromatograms of *Reynoutria* x *bohemica* extract and fractions. Deconvolution of an LC/MS mass chromatogram was carried out by using the Bruker’s Dissect algorithm. Peak numbers are explained in Table 1.

**Figure 3:**
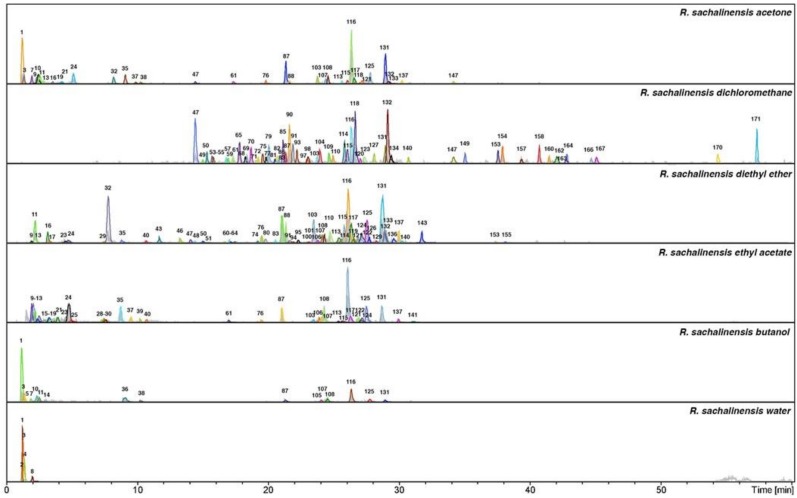
Total ion chromatograms in negative ionization mode and dissect chromatograms of *Reynoutria sachalinensis* extract and fractions. Deconvolution of an LC/MS mass chromatogram was carried out by using the Bruker’s Dissect algorithm. Peak numbers are explained in Table 1.

The LC-MS analysis studies of three *Reynoutria* species (Rj, Rs, Rb) revealed a total of 171 detectable compounds, comprising stilbenes, carbohydrates, procyanidins, flavan-3-ols, anthraquinones, phenylpropanoids, lignin oligomers, hydroxycinnamic acids, naphthalenes and their derivatives. Among the detected chromatographic peaks, 37 remained unassigned and without clear indication of their (phyto)chemical nature and four were tentatively defined as carbohydrates. However, most of the unidentified peaks had UV spectra typical for either hydroxycinnamic (the early eluting) or anthraquinone (late eluting) derivatives. Tentative assignments were carried out based on the MS, MS^2^ and MS^3^ spectra obtained for major *m*/*z* signals recorded in negative ion mode. Further, analysis of UV-vis spectra of compounds and comparison with literature data were used for identification (Figure 1, Figure 2 and Figure 3 and Table 1).

#### 2.1.1. Stilbenoids

Almost all identified stilbenes with characteristic UV spectra with maxima about λ_max_ 220, 305, 320 nm have been previously observed in studied materials [5]. No stilbenes were detected in *R. sachalinensis*. Compounds **20** (piceatannol glucoside) **22** (resveratroloside), **27** (piceid) and **45** (resveratrol), were characterized by HPLC-DAD-ESI-HR-TOF-MS and described in previous article [5]. Only compound **42** with deprotonated molecule at *m*/*z* 389 [M − H]^−^ and fragmentation ion at *m*/*z* 227 characteristic for resveratrol hexoside was noticed for the first time. Compound **42** was observed in small amount in *R. japonica* and *R x. bohemica* dichloromethane or diethyl ether fractions.

#### 2.1.2. Carbohydrates

Deconvolution of an LC/MS mass chromatogram by using the Bruker’s Dissect algorithm allowed to observed several carbohydrates in very similar retention times. Furthermore, the hydrophilic character of compounds and the lack of chromophores confirmed the presence of carbohydrates. Based on deprotonated molecule and fragmentation ions, compounds **1**,**2**,**3** and **4** were described as unknown carbohydrates (Table 1) [10]. Compound **1** exhibited deprotonated molecule at *m*/*z* 341 [M − H]^−^, characteristic for dissacharids e.g., sucrose, which was confirmed by the fragmentation ions at *m*/*z* 179 [M − H-162]^−^, 161 and 143 characteristic for fructose. Compounds **2**, **3** and **4** were more complex but contained the same fragmentation ions at *m*/*z* 341 and 179. More accurate analyses with using different method are needed to identify carbohydrates fully [32]. All apparent carbohydrates were observed in studied *Reynoutria* species water fractions.

#### 2.1.3. Flavan-3-ols and Procyanidins

**Figure 4:**
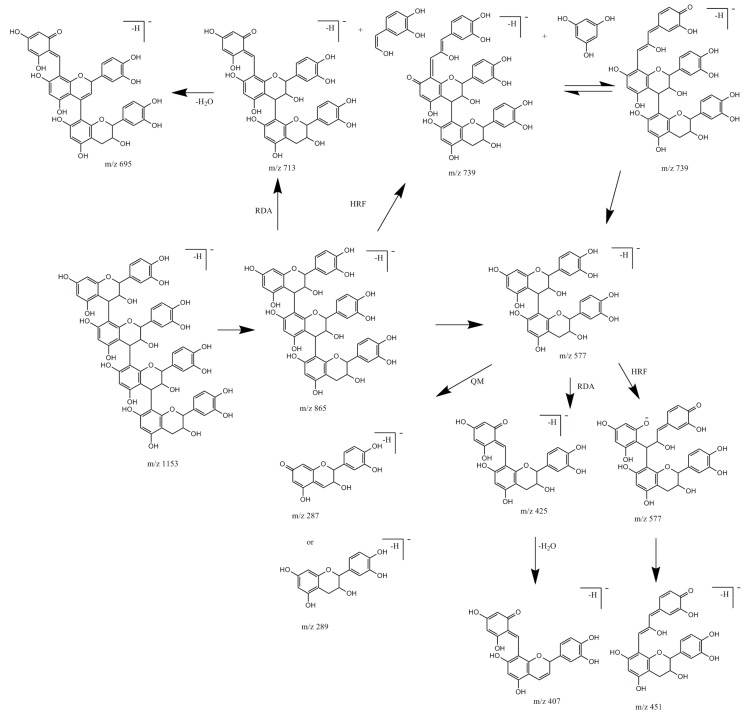
Fragmentation pathways of procyanidins in negative ion mode. RDA, retroDiels-Adler fragmentation; HRF, heterocyclic ring fission; QM—quinone methide cleavage.

B-type procyanidins have different fragmentation patterns than A-type procyanidins and it was used to differentiate procyanidins by the type of linkages between monomeric units [12]. In studied material, there were observed only B-type procyanidins. Identified compounds possessed the same UV spectra characteristic for flavan-3-ols with maxima about λ_max_ 225, 280 and characteristic fragmentation patterns presented in the Figure 4.

Compound **11** was identified as catechin (deprotonated molecule at *m*/*z* 289 [M − H]^−^). Compound **16,** an isomer of **11** with the same molecular mass was identified as epicatechin, both reported earlier in studied species [5] and confirmed with standards. Compounds **7**, **9**, **13**, **33** with a deprotonated molecule at *m*/*z* 577 [M − H]^−^ were identified as procyanidin dimers type B and compounds **10**, **15**, **19**, **21** with deprotonated molecules at *m*/*z* 865 [M − H]^−^ as procyanidin trimers type B [11,12,13].

Compound **25** with deprotonated molecule at *m*/*z* 1153 [M − H]^−^ and with the main product ion at *m*/*z* 865 [M − H-288]^−^ corresponding to procyanidin trimer type B was assigned to procyanidin tetramer type B. Procyanidins with high degree of polymerization, due to the mass range limitations of MS detector were identified by multiple charged ions. Compounds **14** and **26** possessed double- charged ion with signals at *m*/*z* 720 [M − 2H]^2−^ and compound **31**, double-charged ion with signals at *m*/*z* 1008 [M − 2H]^2−^. Taking into account derivative ions (Table 1), characteristic for fragmentation patterns of pentamer and heptamer [12,14,15] they were tentatively assignment to procyanidin pentamers and procyanidin heptamer respectively. According to the literature, compounds **34** and **36** with signals at *m*/*z* 1152 [M − 2H]^2−^ were tentatively assignment to procyanidin octamers [15]. 

Procyanidin gallates were distinguished by their characteristic fragment ion spectra yielding losses of galloyl moiety (−152 Da). Based on the literature [11,13,15] compounds **17**, **24**, **43** were identified as procyanidin dimer monogallates with deprotonated molecule at *m*/*z* 729 [M − H]^−^, and peaks **18**, **23**, **30**, **35**, **40** as procyanidin trimer monogallates with deprotonated molecule at *m*/*z* 1017 [M − H]^−^.

Compound **37** with a deprotonated molecule at *m*/*z* 1305 [M − H]^−^ and a double- charged ion at *m*/*z* 652 [M − 2H]^2−^ as well as with fragmentation patterns characteristic for procyanidins, was tentatively assigned to procyanidin tetramer monogallate [15]. Compounds **29** and **46** revealed deprotonated molecules at *m*/*z* 881 [M − H]^−^ and had characteristic fragmentation pattern for a procyanidin dimer digallate type B. Compound **28** with a deprotonated molecule at *m*/*z* 1169 [M − H]^−^ and a characteristic fragmentation pattern was tentatively assigned to procyanidin trimer digallate [13]. Compound **39** possessed triple-charged ions with signals at *m*/*z* 796 and fragmentation ions characteristic for procyanidin gallate like *m*/*z* 1305 [tetramer gallate] and others (Table 1). It was assigned as procyanidin gallate; probably it is built with more than five monomers and one or more galloyl groups. Similar compound **41** assigned as procyanidin gallate, in their fragmentation possessed ions characteristic for procyanidin gallate, like *m*/*z* 881 for dimer digallate, *m*/*z* 1151 [15] for tetramer procyanidin type A [32], *m*/*z* 441 for catechin monogallate [13] and others (Table 1). Compound **32** with deprotonated molecule at *m*/*z* 441 [M − H]^−^ and confirmed with standards was identified as epicatechin-3-*O*-gallate. 

Procyanidins with degree of polymerization higher than dimers were described for the first time in *R.* x *bohemica.* Whereas most of them were earlier observed in extract of rhizome of *R. japonica* by analyzed it on HPTLC-MS [15]. Using different analytical methods-HPLC-DAD-MS, we confirmed the presence of high order procyanidins, up to decamers in the rhizomes of *R. japonica* and brought new data on the presence of these compounds in other *Reynoutria* species. The presence of a procyanidin trimer digallate has not been reported from any of the studied species before.

#### 2.1.4. Anthraquinones

Compounds **44** and **60** has been previously reported in studied species by using HR-MS analysis [5] and identified as emodin glucoside. Compounds **44** and **60** showed the most abundant product ions at *m*/*z* 269 [M − H-162]^−^ (due to loss of a glucosyl moiety) which was characteristic for emodin. It is supposed that peak **44** corresponds to emodin-1-*O*-glucoside and peak **60** to emodin-8-O-glucoside. Compounds **78**, **120** and **147** were also characterized earlier using a high-resolution time-of-flight MS [5]. Here, the deprotonated molecule at *m/z* 517 [M − H]^−^ for compound **78** showed the most abundant product ion at *m*/*z* 473 [M – H-44]^−^ and product ion at *m*/z 431 [M – H-44-42]^−^ what correspond to fragmentation pattern of emodin-8-*O*-(6′-*O*-malonyl)-glucoside, earlier identified in rhizome of *R. japonica* [11]. In our study, compound **78** was observed also in *R.* x *bohemica*. Compound **120** with a deprotonated molecule at *m/z* 283 [M − H]^−^ showed the most abundant product ion at *m*/*z* 240 and a product ion at *m*/z 268 that correspond to the fragmentation pattern of questin [11]. Questin was observed in all extracts, but only in small amounts in *R. sachalinensis*. The next antraquinone identified in all extracts was emodine (compound **147**), due to its characteristic UV spectrum and fragmentation (a most abundant product ion at *m*/*z* 225 and smaller product ions at *m*/*z* 241 and 182). 

Compound **73** with a deprotonated molecule at *m*/*z* 511 [M − H]^−^ a consecutive loss of SO_3_ (the fragmentation ion at *m*/*z* 431 [M – H-80]^−^) and a glucoside *m*/*z* 269 [M – H-80-162]^−^ led to the formation of an aglycone ion identified as emodin, proved by the diagnostic ions *m*/*z* 241 and 225. Based on the literature [11,20] compound **73** was tentatively identified as emodin-*O*-(sulfonyl)-glucoside observed only in the *R. japonica* butanol fraction. 

**Figure 5:**
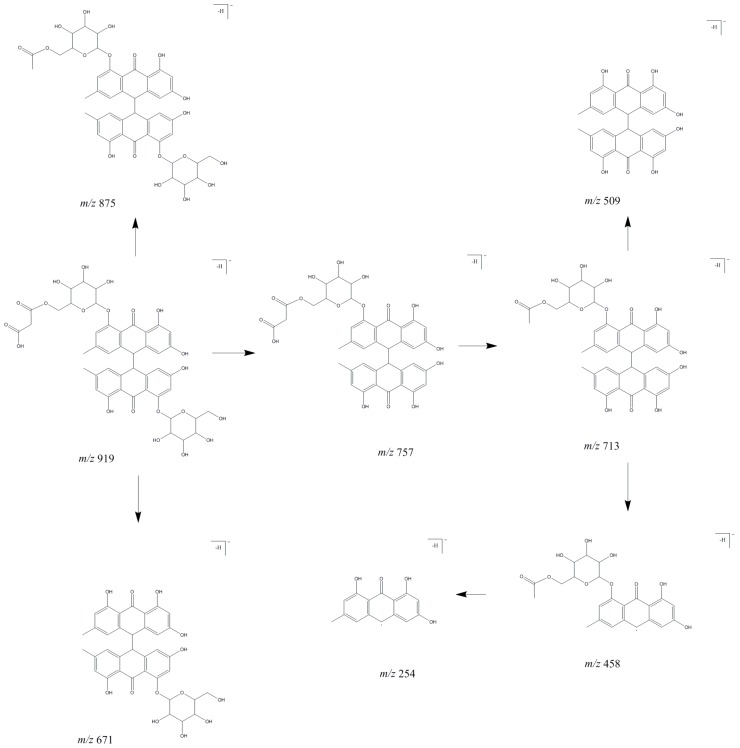
Proposed fragmentation pathway for peaks 66, 92, and 96.

Compounds **66**, **92** and **96** were observed in studied species for the first time, all of them exhibited the same deprotonated molecule at *m*/*z* 919 [M − H]^−^ and similar fragment ions despite little difference in intensity. The proposed fragmentation map is shown in Figure 5.

Malonyl-substituted type glucosides are widely found in *Fallopia multiflora* and *Rheum* plants. Due to a lack of standard compounds and the fact no dianthrones have been found earlier in *R. japonica*, *R.* x *bohemica* and *R. sachalinensis*, the structural characterization of the new dianthrone glycosides was referred to the literature on *Rheum* genus plants and *F. multiflora* [21,33,34] in which the MS fragmentation behavior of dianthrone glycosides was well described. Malonyl-substitution of dianthrone glycosides was earlier described in *F. multiflora* [21]. In our study, compounds **66**, **92** and **96** tentatively assigned as emodin bianthrone-hexose-(malonic acid)-hexose were observed only in *R.* x *bohemica* extract and *R.* x *bohemica* butanol fraction. 

Compounds **84**, **102**, **111** exhibited the same deprotonated molecule at *m*/*z* 933 [M − H]^−^ and the same most abundant fragment ion at *m*/*z* 889 [M – H-44]^−^ due to loss of CO_2_ and fragment ion at *m*/*z* 727 [M – H-44-162]^−^ produced by a cleavage of a glucosidic bond. The deprotonated molecule at *m*/*z* 933 [M − H]^−^ differed from those of compounds **66**, **92**, **96** by 14 Da, which corresponds to a methyl moiety.

Based on their fragmentation patterns (similar to that presented in Figure 5, but with the addition of a methyl moiety) and literature, these compounds were tentatively identified as methyl derivatives of emodin bianthrone-hexose-(malonic acid)-hexose [21] which were observed as small peaks only in *R.* x *bohemica* butanol fraction. Compounds **89**, **112**, **130** with the same deprotonated molecules at *m*/*z* 1019 [M − H]^−^ and fragmentation ions like described above: *m*/*z* 889, 727, 458, suggest the presence of methyl derivatives of emodin bianthrone-hexose-(acetyl)-hexose. The mentioned compounds are fragmented to ions at *m*/*z* 975 [M − H-44]^−^ due to loss of CO_2_, the most abundant product ion is at *m*/*z* 931 [M − H-44 × 2]^−^ due to the loss of a second CO_2_. However, because of the many possible structures of compounds **89**, **112**, **130**, they were described as derivatives of emodin bianthrone-di-hexose.

The exact structure of these compounds requires detailed research. Compounds **89**, **112**, **130** were observed only in *R.* x *bohemica* extract and butanol fraction. Compounds **128**, **142**, **144** showed the same deprotonated molecule at *m*/*z* 757 [M − H]^−^. Due to the characteristic fragmentation ions at *m*/*z* 713, 509, 458 (Figure 5), they were tentatively identified as emodin bianthrone-hexose-malonic acids. These compounds were mainly observed in *R.* x *bohemica* extract and fractions and as a small peak in the *R. japonica* diethyl ether fraction. None of them were observed in *R. sachalinensis*. 

Compounds **148**, **150**, **151**, **152** exhibited the same deprotonated molecule at *m*/*z* 771 [M − H]^−^, that differed from peaks described above for **128**, **142**, **144** by 14 Da, what could correspond to a methyl moiety loss. The most abundant product ion at *m*/*z* 727 [M − H-44]^−^ was observed due to the loss of CO_2_. The product ion at *m*/*z* 502 [M − H-269]^−^ was caused by the 10–10′ homolytic cleavage of anthrone and the product ion at *m*/*z* 458 [M − H-44-269]^−^ by cleavage of anthrone and loss of CO_2_. Peaks were tentatively identified as a methyl derivative of emodin bianthrone-hexose-malonic acid. Peaks were observed only in *R.* x *bohemica* extract and fractions. 

Compounds **67** and **105** showed the same deprotonated molecule at *m*/*z* 1005 [M − H]^−^. Fragmentation ion at *m*/*z* 757 [M − H-248]^−^ could represent emodin bianthrone-hexose-malonic acid as confirmed by subsequent fragmentation ions: the most abundant ion at *m*/*z* 713 [M − H-248-44]^−^ due to the loss of CO_2_, product ion at at *m*/*z* 458 [M − H-248-44-255]^−^ by cleavage of anthrone (Figure 5). Moreover, a deprotonated molecule at *m*/*z* 1005 [M − H]^−^ after loss of CO_2_ created an ion at *m*/*z* 961 [M − H-44]^−^ and after more loss of CO_2_ an ion at *m*/*z* 917 [M − H-44 × 2]^−^ was created. Due to the many possible structures of compounds **67** and **105**, they were described as derivative of emodin bianthrone-hexose-malonic acid. The exact structure of these compounds requires more detailed research. Compounds were observed in the butanol fractions of all studied *Reynoutria* species.

Compound **138** observed in extract of *R. japonica* and *R.* x *bohemica* exhibited a deprotonated molecule at *m*/*z* 671 [M − H]^−^ and a product ion at *m*/*z* 653 [M − H-18]^−^, due to the loss of H_2_O moiety, a product ion at *m*/*z* 509 [M − H-162]^−^ by loss of a hexosyl moiety, and the most abundant product ion at *m*/*z* 416 [M − H-255]^−^ caused by the 10–10′ homolytic cleavage of anthrone and the product ion at *m*/*z* 254 [M − H-255-162]^−^ by cleavage of anthrone and hexosyl moieties (Figure 5). Based on fragmentation pattern and literature, compound **138** was tentatively identified as emodin bianthrone-hexose [21]. Compounds **145** and **146** with the same deprotonated molecules at *m*/*z* 685 [M − H]^−^ differed from peak **138** by 14 Da what corresponds to loss of a methyl moiety. What is more, compounds **145** and **146** exhibited product ions at *m*/*z* 416 and 254, described above. These compounds were tentatively identified as methyl derivatives of emodin bianthrone-hexose. The compounds were observed only in *R.* x *bohemica* dichloromethane and diethyl ether fractions.

Compounds **161** and **165** with the same deprotonated molecules at *m*/*z* 509 [M − H]^−^, fragmentation ions at *m*/*z* 491 [M − H-18]^−^, due to the loss of H_2_O and fragmentation ions at *m*/*z* 254 [M − H-255]^−^ caused by the 10–10′ homolytic cleavage of anthrone were tentatively identified as emodin bianthrones (Figure 5). The compounds were observed in *R. japonica* and in *R.* x *bohemica* fractions.

Compound **169** exhibited the same UV spectra with a maximum about λ_max_ 220, 278, 360 nm, like compounds **161** and **165**. Deprotonated molecule at *m*/*z* 523 [M − H]^−^ fragmented to ion at *m*/*z* 254 [M − H-269]^−^ caused by the 10–10′ homolytic cleavage of anthrone. Compound **169** differed from peak **161** and **165** by 14 Da that corresponds to a methyl moiety. The compound was tentatively identified as methyl derivative of emodin bianthrone.

#### 2.1.5. Phenylpropanoid Disaccharide Esters

Phenylpropanoid-derived disaccharide esters possess a sucrose core carrying a varying number of *O*-substituents, including phenylpropanoid, acetyl, benzoyl, *p*-methoxybenzoyl, and *p*-hydroxy-benzoyl groups. Peaks **48**, **74**, **76**, **87**, **101**, **88**, **100**, **103**, **110**, **115**, **113**, **116**, **124**, **117**, **125**, **129**, **131**, **136** corresponding to acethyl lapathoside D, lapathoside C and isomers, e.g., hydropiperoside A [28], hydropiperoside and isomer, (3,6-*O*-di-*p*-coumaroyl)-β-fructofuranosyl-(2→1)-(2′-*O*-acetyl-6′-*O*-feruloyl)-β-glucopyranoside and an isomer, vanicoside C, tatariside B and an isomer, vanicoside B and isomers, lapathoside A, dihydroferuloyl vanicoside B and an isomer, vanicoside A and an isomer (Table 1) were observed in the studied species previously [5,16,23]. The remaining phenylpropanoid disaccharide esters were detected in the present study for the first time. The identified phenylpropanoid-derived disaccharide esters possessed the same UV-Vis spectra characteristic for flavan-3-ols with maxima at about λ_max_ = 220, 290, 315 nm. Compounds **62**, **63** and **80** possessed the same deprotonated molecules at *m*/*z* 717 [M − H]^−^ and similar fragmentation patterns with the most abundant ions at *m*/*z* 571 [M − H-146]^−^ caused by a loss of deoxyhexosyl, which gives in the MS^3^ analysis similar ions with the most abundant one at *m*/*z* 529 [M − H-42]^−^ produced by loss of acetyl. According to [19] these compounds were tentatively identified as tatariside E and isomers. Compounds **83** and **95** with deprotonated molecules at *m*/*z* 759 [M − H]^−^ and with characteristic fragmentation patterns (Table 1), were tentatively assigned as tatariside A and an isomer [19]. Both tatariside E and tatariside A were previously isolated from *Fagopyrum tataricum* [19]. Compound **119** observed in the diethyl ether fraction of *R. sachalinensis* and *R.* x *bohemica* with a deprotonated molecule at *m*/*z* 1015 [M − H]^−^ and characteristic fragments was assigned as lapathoside B, isolated and described earlier from *Polygonum lapathifolium* [26]. Compound **132** with a deprotonated molecule at *m*/*z* 935 [M − H]^−^ was identified as tatariside C, earlier isolated from *Fagopyrum tataricum* [19,27]. Compound **132** had an additional acetyl group relative to tatariside B. Fragmentation ions of compound **132** were characteristic for tatariside B, e.g., *m*/*z* 893 and others (Table 1). Compound **133**, observed in all studied species, with a deprotonated molecule at *m*/*z* 1027 [M − H]^−^ was tentatively identified as hydropiperoside B, isolated for the first time from *Polygonum hydropiper* [28]. The deprotonated molecule at *m*/*z* 1027 gave a product ion at *m*/*z* 985, which corresponds to the loss of the acetyl group from hydropiperoside B and was the same as the deprotonated molecule of lapathoside A. Similar compound **143** with a deprotonated molecule at *m*/*z* 1039 [M − H]^−^ was identified as vanicoside E, after losing the acetyl group, giving a product fragmentation ion at *m*/*z* 997 [M − H-42]^−^, characteristic for the deprotonated vanicoside A molecule [28]. Vanicoside E was observed in the diethyl ether fraction of *R. sachalinensis* and in small amounts in *R.* x *bohemica.* Compounds **107** and **108** with deprotonated molecules at *m*/*z* 1151 [M − H]^−^ were earlier observed in rhizomes of *R. sachalinensis* [23] and were described as undefined phenylpropanoid glucoside. Compounds **107**, **108** gave fragmentation ions characteristic for dihydroferuloyl vanicoside B at *m*/*z* 1133 and for vanicoside B, *m*/*z* 955, *m*/*z* 809, and were observed in all studied species. Compound **106** with a deprotonated molecule at *m*/*z* 1181 [M − H]^−^, observed in small amounts only in the diethyl ether fraction of *R. sachalinensis* and *R.* x *bohemica,* was noticed there for the first time. It has been described as a disaccharide phenylpropanoid ester derivative due to its UV-Vis spectrum and fragmentation ions, characteristic for this group of compounds (Table 1). Compounds **121**, **122** and **137** with deprotonated molecules at *m*/*z* 1193, 1163 and 1175, respectively, were observed for the first time in the studied species and were described as disaccharide ester derivatives of phenylpropanoids due to fragmentation ions such as *m*/*z* 997 (vanicoside A), *m*/*z* 955 (vanicoside B). Compound **141** which was observed only in the ethyl acetate fraction of *R. sachalinensis*, possessed a triple-charged ion with a signal at *m*/*z* 954, but also fragmentation ions at *m*/*z* 809 characteristic of lapathoside C, *m*/*z* 779 characteristic of hydropiperoside, as well an UV-Vis spectrum with maxima at λ_max_ 220, 290, 315 nm and this compound was described as a disaccharide ester derivative of phenylpropanoid. Compound **50** observed in *R.sachalinensis* and *R.* x *bohemica* fractions was tentatively assigned as a disaccharide ester phenylpropanoid derivative because of its fragmentation ions at *m*/*z* 613, 571, similar to the fragmentation ions of tatariside A (compound **83**). Compound **94** was tentatively assigned as a phenylpropanoid disaccharide ester derivative because of its UV-Vis spectrum similarity and fragmentation ions at *m*/*z* 851 ((3,6-*O*-di-*p*-coumaroyl)-β-fructofuranosyl-(2→1)-(2′-*O*-acetyl-6′-*O*-feruloyl)-β-glucopyranoside) and ions at *m*/*z* 633, 453 similar to the fragmentation ions of hydropiperoside.

#### 2.1.6. Lignin Oligomers

Compounds tentatively identified as lignin oligomers (LOs) were observed in the dichloromethane fractions of studied *Reynoutria* species. All LOs were seen in the studied raw materials for the first time. Identification was made based on the fragmentation pattern of the LOs and the UV/VIS spectrum and comparisons with the literature.

**Figure 6:**
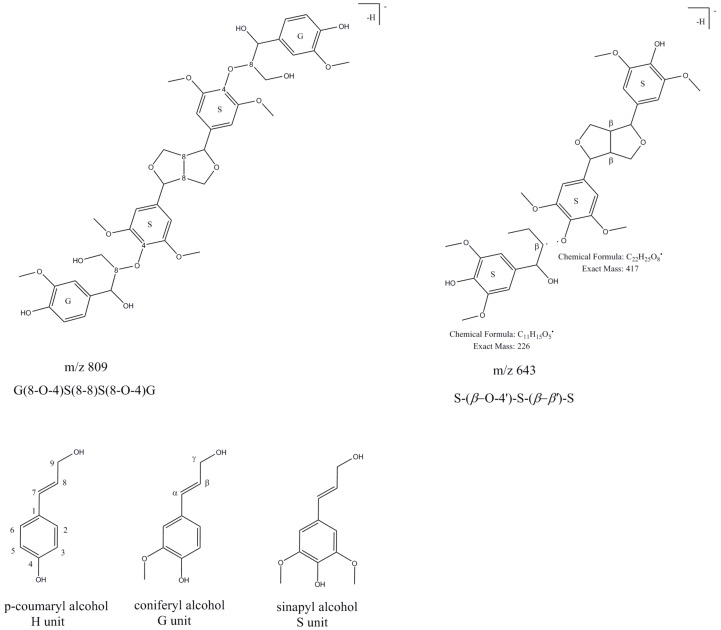
Lignin oligomers.

Coniferyl alcohol (G unit), sinapyl alcohol (S unit) and *p*-coumaryl alcohol (H unit) are linked covalently, forming ether, ester and carbon–carbon bonds, which repeat to provide the great complexity of lignin [25]. The degree of polymerization in natural lignin is difficult to measure because it is supposed that it fragments during extraction [35]. Therefore, lignin fragments, oligomers of lignin, are the species most often identified in plant extracts. Compounds **71** and **72** were observed only in the dichloromethane fraction of *R. sachalinensis* and were identified based on fragmentation patterns described in [25]. The deprotonated molecule of compound **71** at *m*/*z* 643 [M − H]^−^ was tentatively identified as a trimer of lignin β-*O*-4-linked S unit with syringaresinol [*S*-(β-*O*-4′)-*S*-(β-β′)-*S*] due to its fragmentation pattern which corresponds to that described by Evtuguin et al. [22]. The characteristic and most abundant fragmentation ion at *m*/*z* 417 corresponds to deprotonated syringaresinol (Figure 6).

Compound **72** with a deprotonated molecule at *m*/*z* 869 differs by 226 Da from compound **71**, what correspond to the syringyl phenylpropane unit. Based on the fragmentation pattern, which was similar to that of peak **71** and based on [22], peak **72** was assigned as tetramer lignin, *S*-(8-*O*-4′)-*S*-(8-*O*-4′)-S-(8-8′)-*S*. Compound **81**, observed as very small peak in the dichloromethane fraction of *R. sachalinensis* was described as a derivative of lignin-*S*(8–8)*S*. UV/VIS spectrum (λ_max_ at 220 and 280 nm) and its fragmentation ions at *m*/*z* 417 and 387 (-CH_2_O), suggest that compound **81** is composed of syringaresinol. Compounds **79**, **85** and **93** with the same deprotonated molecules at *m*/*z* 809 [M − H]^−^ and fragmentation ions were observed in all dichloromethane fractions of *Reynoutria* species. The deprotonated molecule at *m*/*z* 809 [M − H]^−^ suggested a tetrameric compound structure, composed of two **G** and two **S** units (Figure 6). MS/MS spectral peaks at *m*/*z* 791 (-H_2_O), 773 (-2H_2_O), 761 (-CH_2_O and H_2_O), 743 (-CH_2_O and 2H_2_O) indicated the presence of two *β*-aryl ether units and a fragmentation ion at *m*/*z* 417 corresponds to deprotonated syringaresinol [24]. This MS and MS/MS spectrum was similar to the spectrum of oligolignol: G(8-*O*-4)S(8-8)S(8-*O*-4)G [24] called hedyotisol [36].

#### 2.1.7. Other Hydroxycinnamic Acid Derivatives

**Figure 7:**
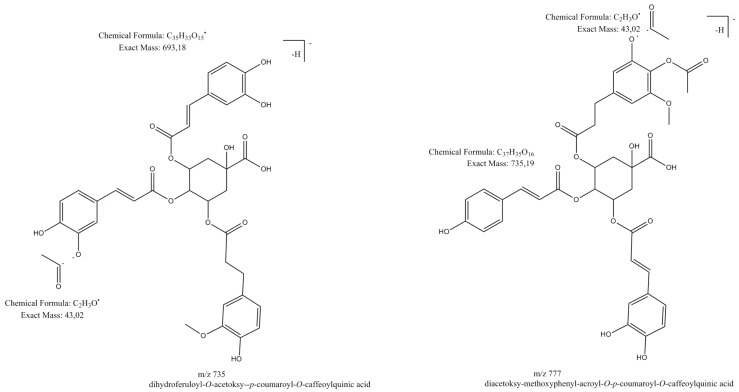
Examples of hydroxycinnamic acids esters.

The deprotonated molecule at *m/z* 735 [M − H]^−^ for compound **61** was observed in all extracts. The peak showed a product ion at *m*/*z* 693 [M − H-42]^−^, due to the loss of an acetyl moiety. The most abundant product ion at *m*/*z* 559 was due to the loss of a feruloyl or isoferuloyl group. The fragmentation pattern showed ions at *m*/*z* 499 and 337, which were characterized as a *p*-coumarylquinic acid moiety. Based on fragmentation pattern and comparisons with the literature, compound **61** was tentatively assigned as dihydroksyferuloyl-*O*-acetoxy-*p*-coumaroyl-*O*-caffeoylquinic acid [18] (Figure 7).

Deprotonated molecules at *m/z* 777 [M − H]^−^ (Figure 7) for compounds **65**, **70** and **75** showed the most abundant product ion at *m*/*z* 735 [M − H-42]^−^, due to the loss of an acetyl moiety, a product ion at *m*/*z* 693 [M − H-42 × 2]^−^ due to the loss of the next acetyl moiety, product ions at *m*/*z* 717 [M − H-42-18]^−^ due to the loss of an acetyl moiety and H_2_O. Fragmentation ions at *m*/*z* 499 and 337 are characterized as *p*-coumarylquinic acid moieties [37,38,39]. Based on the fragmentation pattern and comparisons with the literature, compounds **65**, **70**, **75** were tentatively assigned as (diacetoxy-methoxyphenyl)acroyl-*O*-*p*-coumaroyl-*O*-caffeoylquinic acid and its isomers [18]. 

Compounds **86**, **90**, **98**, **99**, **104** showed deprotonated moleculee at *m/z* 819 [M − H]^−^ and similar fragmentation ions like compounds **65**, **70**, **75**, for example the most abundant product ion at *m*/*z* 777 [M − H-42]^−^ due to the loss of an acetyl group. Compounds **86**, **90**, **98**, **99**, **104** were tentatively assigned as acetyl derivatives of (diacetoxy-methoxyphenyl)acroyl-*O*-*p*-coumaroyl-*O*-caffeoylquinic acids [18]. The most abundant peak **118**, observed in all dichloromethane fractions and peaks **123**, **127** with deprotonated molecules at *m*/*z* 861 [M − H]^−^ were described as diacetyl derivatives of (diacetoxy-methoxyphenyl)acroyl-*O*-*p*-coumaroyl-*O*-caffeoylquinic acid. The product ion at *m*/*z* 819 [M − H-42]^−^ due to the loss of acetyl moiety and the rest of the fragmentation ions were similar to earlier described hydroxycinnamic acid derivatives. Compound **134** observed in the dichloromethane fraction of *R. sahalinensis* with a deprotonated molecule at *m*/*z* 965 [M − H]^−^ and compound **135** observed in the dichloromethane fraction of *R.* x *bohemica* with a deprotonated molecule at *m*/*z* 995 [M − H]^−^ due to the more complex structure was described as derivatives of (diacetoxy-methoxyphenyl)acroyl-*O*-*p*-coumaroyl-*O*-caffeoylquinic acid. However, it can be assumed that compound **134** is a coumaroyl or deohexosyl derivative of compound **86** or its isomers, due to the loss of the moiety at *m*/*z* 146 and a product ion at *m*/*z* 819 [M − H-146]^−^. The fragmentation of the product ion at *m*/*z* 819 gave product ions which were similar to those of compound **86**, whereas fragmentation of compound **135** gave the most abundant product ion at *m*/*z* 819 [M − H-176]^−^ due to the loss of feruloyl or oxyhexosyl moiety.

#### 2.1.8. Naphthalene Derivatives

Compound **56** was characterized by HPLC-DAD-HR-MS analysis in a previous article as torachrysone glucoside [5]. Peak **56** showed deprotonated molecule at *m/z* 407 [M − H]^−^ and product ion at *m*/*z* 245 [M − H-162]^−^ by cleavage of a glucosidic bond and characteristic for the torachrysone fragmentation ion at *m*/*z* 230 [M − H-162-15]^−^. Torachrysone glucoside was noticed in the acetone extract and dichloromethane fractions of *R. japonica* and *R.* x *bohemica.*

#### 2.1.9. Other Compounds

Compound **47** with a deprotonated molecule at *m*/*z* 312 [M − H]^−^ was earlier identified as *N*-*trans*-feruloyltyramine by HPLC-DAD-HR-MS analysis and described in our previous article [5]. Using a different analytical instrument, based on compound MS, MS^2^ and MS^3^ spectra, its identity was confirmed. Moreover, compound **49** exhibited a similar UV/VIS spectrum (λ_max_ at 220, 280, 323 nm) and fragmentation pattern to compound **47** (*m*/*z* 297, 178, 135) and differed from compound **47** by 30 Da, which could result from methoxylation. Based on the fragmentation ions and reference [17] compound **49** was tentatively assigned as *N*-feruloylmethoxytyramine, observed in the studied plants for the first time. 

Compound **51**, because of its deprotonated molecule at *m*/*z* 287 and product ion at *m*/*z* 269, the most abundant product ion at *m*/*z* 151 and product ion at *m*/*z* 135, 125, 107 was tentatively identified as cyanidin [18]. Unfortunately due to the fact the UV–vis spectra was recorded in the range of 200–450 nm, it was impossible to get all the maximum spectra of this compound to confirm the assumption. The compound was noticed in fractions of *R.* x *bohemica* and *R. sachalinensis.*

Compound **5** observed in fractions of *R.* x *bohemica* and *R. sachalinensis* with a deprotonated molecule at *m*/*z* 331 [M − H]^−^ and the most abundant product ion at *m*/*z* 169 [M − H-162]^−^ due to glucosidic bond cleavage was tentatively, based on [11], described as galloyl glucose, earlier observed in *R. japonica* rhizomes.

Compound **159** showed a deprotonated molecule at *m/z* 755 [M − H]^−^, product ion at *m*/*z* 593 [M − H-162]^−^ by cleavage of a glucosidic bond, the most abundant product ion at *m*/*z* 575 [M − H-162-18]^−^ due to the loss of a glucosyl moiety and H_2_O, product ion at *m*/*z* 431 [M − H-162 × 2]^−^ produced by cleavage of two glucosidic bonds. The next fragmentation of the product ion at *m*/*z* 575 showed that the most abundant fragment ion was *m*/*z* 431, what together with the rest of the fragmentation ions and characteristic UV/VIS spectrum (λ_max_ at 269, 333 nm) suggested that peak **159** could be isovitexin or vitexin diglucoside [29,30]. It was observed only in the dichloromethane fraction of *R.* x *bohemica.* It was noticed for the first time in this species.

Compound **170** because of its lipophilic character and deprotonated molecule at *m/z* 277 [M − H]^−^, product ion at *m*/*z* 259 [M − H-18]^−^ due to the loss of H_2_O and the most abundant product ion at *m*/*z* 233 [M − H-44]^−^ due to loss of CO_2_ was tentatively assigned as α-carboxyethylhydroxychroman [31]. It was observed in the dichloromethane fractions of *R.* x *bohemica* and *R.sachalinesis.*

### 2.2. Antioxidant Activities and Polyphenols Content

Results of bioactivity screening of all 18 extracts and fractions are presented in Table 2.

All studied acetone extracts demonstrated high ability to scavenge the 2,2′-diphenyl-picrylhydrazyl radical, comparable to ascorbic acid. Fractionation of extracts allowed us to obtained fractions like the ethyl acetate one with even stronger stable radical scavenging properties. 

High ability to scavenge stable radicals was associated with high amount of polyphenols, especially tannins in the studied extract and fractions (Table 3), what was demonstrated by the Spearman Rank Order Correlation in Table 4.

**Figure 8:**
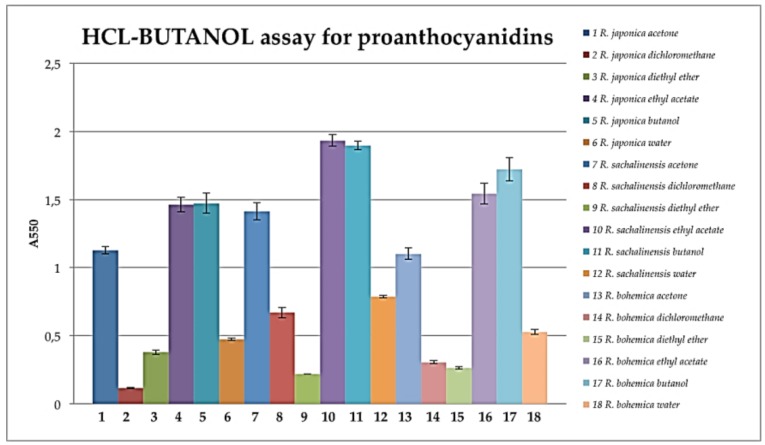
Proanthocyanidins quantified spectrometrically by absorbance at 550 nm in extracts and fractions. Data were expressed as mean ± SD, performed in at least three independent experiments, assayed in triplicate.

These results are in accordance with the above presented phytochemistry of extracts and fractions, where the most antioxidant active ethyl acetate fractions contained numerous polyphenols including procyanidins (Figure 1, Figure 2 and Figure 3, Table 1). Ethyl acetate fractions, which were the richest in polyphenols and tannins, exhibited also the highest capacity to reduce metal ions (phosphomolybdenum reduction assay) and to prevent the oxidation of linoleic acid. Diethyl ether and butanol fractions of studied species exhibited slightly weaker antioxidant activity, however they also contained significantly lower contents of total polyphenols and tannins (except *R.* x *bohemica* diethyl ether fraction, where the differences with the ethyl acetate fraction were not significant). Because the results indicated the big impact of tannins on antioxidant activity, what was according with phytochemical analysis, we decided to check the amount of procyanidins in the studied extracts and fractions using the acid butanol method (Bate-Smith method) [40] and DMACA-HCl assay. Results, presented in Figure 8, revealed that ethyl acetate and butanol fractions contained the highest amount of proanthocyanidins, whereas *R. sachalinensis* ethyl acetate and butanol fractions contained significantly higher amount proanthocyanidins than others.

Although the acetone extract of *R. sachalinensis* contained the highest amount of proanthocyanidins, the diethyl ether fraction contained the lowest amounts compared to diethyl ether fractions from other species. This indicates different fractionation efficiency, which could be affected by differences in the composition of the mixtures or different individual structures of the separating compounds. The content of proanthocyanidins in the butanol fractions is very similar to the content in the ethyl acetate fractions of the studied species, despite the fact that the Folin-Ciocalteu assay showed significantly less content of tannins in the butanol fractions than in the ethyl acetate fractions.

**Figure 9:**
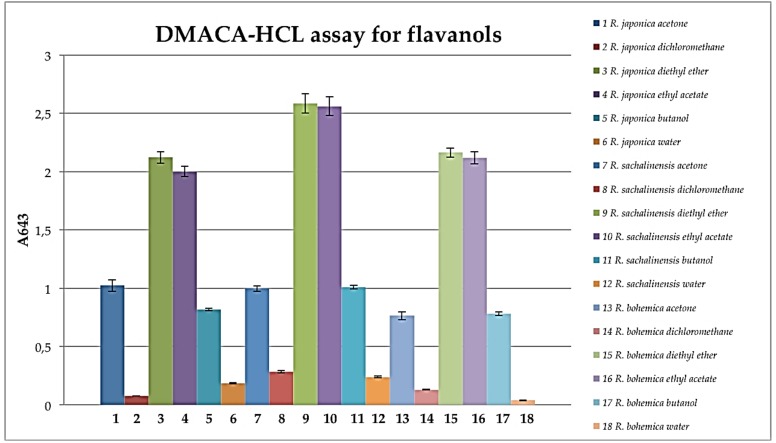
Flavanols quantified spectrophotometrically for absorbance at 643 nm in extracts and fractions. Data were expressed as mean ± SD, performed in at least three independent experiments, assayed in triplicate.

It is important to mention that the acid butanol method we used to measure the amount of proanthocyanidins involves depolymerization of the polymer of proanthocyanidins in acid and conversion of the monomers to anthocyanidins, which were spectrophotometrically quantified. Based on our results, we assumed that there are more proanthocyanidins with higher degree of polymerization in the butanol fractions of all studied species than in the ethyl acetate fractions. This assumption agrees with LC-MS analysis in which compounds putatively identified as procyanidin heptamer and octamer were noticed mainly in butanol fractions of studied species. The results from the DMACA assay indicated that in diethyl ether and ethyl acetate fractions are significantly more flavanols than in butanol fractions (Figure 9).

4-Dimethylaminocinnamaldehyde (DMACA) reacts with *m*-diphenols to form coloured carbonium ions in acid and this reaction is utilized for the assay of flavanols, because the A-rings of flavanols have *m*-diphenol functionalities [41]. The DMACA reaction affects the C8 position of the A-ring and reacts only with the terminal units of a proanthocyanidins. In this assay, it does not matter how many monomers a proanthocyanidin molecule is made of, but it indicates how many free C8 positions it has. The results agree with the assumption that in the ethyl acetate fractions contain more proanthocyanidin molecules than the butanol ones, but they are made up of fewer monomers. High results in the DMACA assay in diethyl ether fractions may be due to a high content of flavanols other than procyanidins, such as catechin, epicatechin or epicatechin-3-*O*-gallate what is in accordance with chromatographic analysis of these fractions.

**Figure 10:**
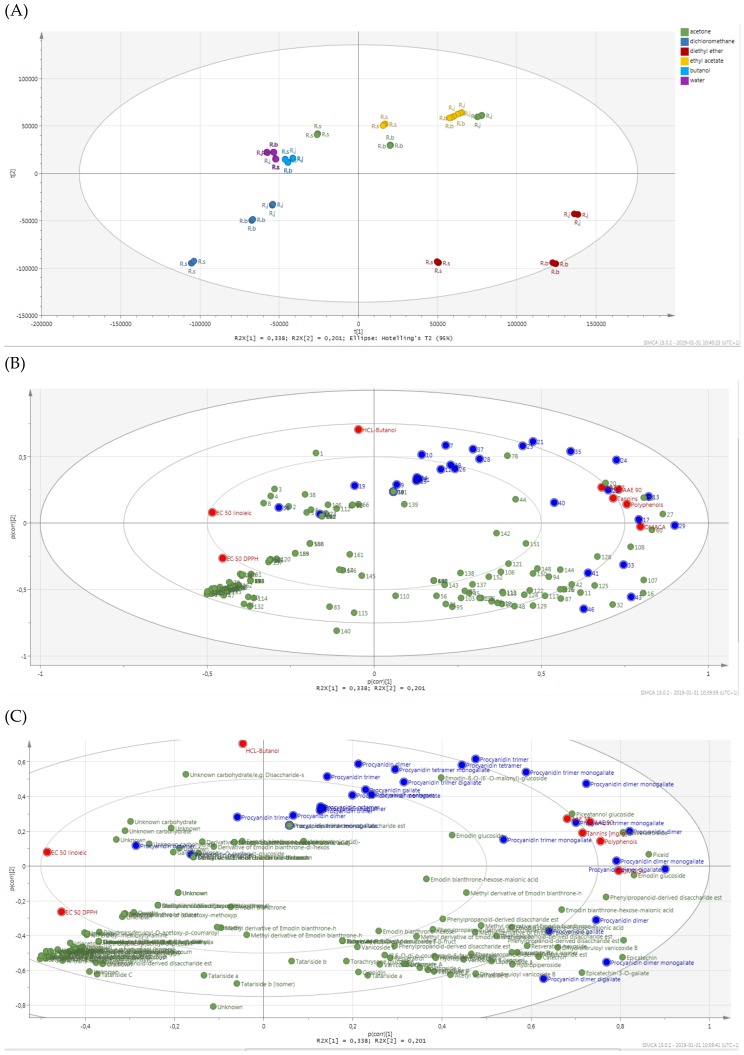
DPPH free radical scavenging activity of vanicoside A, vanicoside B, *R. sachalinensis* acetone extract and *R. sachalinensis* ethyl acetate fraction with range of concentrations. SC% percentage of scavenging activity on DPPH radical. The absorbance at 517 nm was measured after 30 min.

In order to observe relationships between the individual compounds present in the fractions and antioxidant activity, we used chemometric analyses. The principal component analysis (PCA) allowed exploratory analyses of the data which included the results of antioxidant tests and the LC-MS data (peak area of compounds), summarizing the multidimensional data in an intelligible way to detect the underlying characteristics and structures of the data (Figure 10).

The visualization of the PCA scores plot shows similarities/dissimilarities between (explained by principal component 1 (PC1)) and within (explained by PC2) the sample clusters. On the PCA score plot all ethyl acetate and diethyl ether fractions with the most antioxidant activity as well as *R.j* and *R.b* acetone extracts were located on the right side of the plot. According to the loading plot for this differentiation, compounds located mostly in the right plot are responsible, such as procyanidins (mainly **13**- procyanidin dimer, **17**- procyanidin dimer monogallate, **29**- procyanidin dimer digallate), stilbenes (mainly **20**- piceatannol glucoside, **22**- resveratrolside, **27**- piceid), emodin glucoside (**60**), as well as almost all performed assays (without HCl-butanol). Dissimilarities between the ethyl acetate and diethyl ether fractions distributed in the third and fourth quadrant are explained by PC2. According to the loading plot the biggest impact on the created ethyl acetate and acetone cluster in the third quadrant had procyanidins and HCl-butanol, whereas for diethyl ether cluster formation phenylpropanoid disaccharide esters were relevant, as well as catechin (**11**), epicatechin (**16**), epicatechin-3-*O*-gallate (**32**) and some procyanidins (compounds **33**, **41**, **43**, **46**). The PCA score plot reveals the difference between *R. sachalinensis* and the more similar to each other *R. japonica* and *R.* x *bohemica.* According to the loading plot, in the case of the acetone extract, ethyl acetate and diethyl ether fractions, dissimilarities are the result of a smaller contribution of PC1, which is in accordance with the phytochemical analysis, where, among others, no stilbenes were observed in the *R. sachalinensis* extract and fractions. Moreover, the loading plot revealed a high correlation of the performed assays (except HCl-butanol) to each other, which agrees with the results in Table 4. Located on the left side of the plot the results from the DPPH assay and linoleic acid peroxidation assay are due to the usage of EC_50_ as an activity measure (i.e., a lower value of the parameter means a higher activity). Considering the location of AAE 37 and AAE 90 on the loading plot, it can be suggested that there were correlations with procyanidins and some stilbene compounds relatively close located to the AAE 37 and AAE 90 points. In the case of the DPPH assay, a strong correlation is seen mainly with procyanidins, located in the third quadrant of the loading plot, close to the line extension running from the DPPH EC_50_ point through point 0. Similarly in the case of the EC_50_ values in the linoleic acid peroxidation assay, the correlation seems to be strong also with some of the phenylpropanoid disaccharide esters found in the fourth quadrant of loading plot. These assumptions are consistent with the results presented in Table 5, which shows the strength of the correlations of compounds with antioxidant assays.

**Figure 11:**
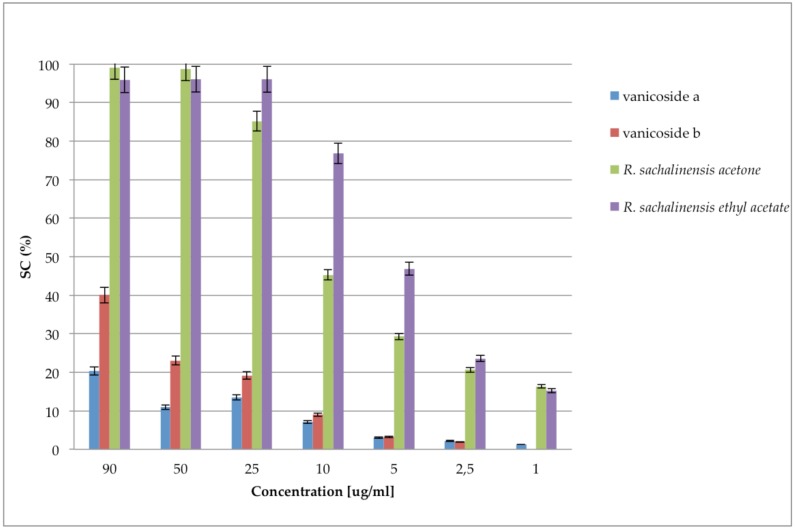
Principal components analysis (PCA) plots indicating the general grouping of the variables in the data sets of extracts (green-acetone) and fractions (blue-dichloromethane, red-diethyl ether, yellow-ethyl acetate, blue-butanol, purple-water) from R.j-*Reynoutria japonica*, R.b-*Reynoutria* x *bohemica*, R.s-*Reynoutria sachalinensis* in three independent experiments. (**A**) The PCA score plot of the LC-MS data and antioxidant assay illustrates the general clustering of the variables. The scores plot was computed using the first two principal components (PC1 vs. PC2). The circle in the score plot represents Hoteling’s T2 with 95% confidence interval. R2X(cum) = 0.911, Q2 (cum) = 0.693 for 7 components. (**B**) Loading plot of PCA results obtained from LC-MS data and antioxidant assay. Numbers represent the compounds listed in Table 1. Blue points represents procyanidins, red—antioxidant tests, green-all compounds without procyanidins. (**C**) Enlarged image of the loading plot of PCA with named compounds.

The presented statistical analyses show that the high antioxidant activity of fractions and extracts was significantly influenced by procyanidins. Interestingly, stilbenes occurring in a significant amount in the *R. japonica* and *R.* x *bohemica* extract and fractions and phenylpropanoid disaccharide esters, especially vanicoside A and B, occurring in a significant amount in the *R. sachalinensis* extract and fractions turned out to have less influence on antioxidant activity of the studied samples. Considering that the *R. sachalinensis* ethyl acetate fraction with the most antioxidant activity contained almost only procyanidins and phenylpropanoid disaccharide esters, especially a high amount of vanicoside A and B, we decided to check the DPPH free radical scavenging activity of isolated vanicosides A and B to find out to what extent they affect the fraction activity. Results from the DPPH free radical scavenging activity of vanicoside A, vanicoside B, presented in Figure 11, revealed significantly weaker activity of the tested compounds in relation to the acetone and ethyl acetate *R. sachalinensis* fraction. Thus other compounds must influence the strong fraction activity.

Fan et al. [23] measured the free radical scavenging activity of four phenylpropanoid-derived disaccharide esters obtained from stems of *R. sachalinensis*, which scavenging increased as follows: vanicoside B < hydropiperoside < lapathoside C < lapathoside D, whereas 95 μg/mL of vanicoside B demonstrated scavenging about 32% of DPPH (what was similar to our result) and 95 μg/mL of lapathoside D scavenging about 75% of DPPH. Taking the above results into account, even the strongest scavenger activity of phenylpropanoid-derived disaccharide esters does not explain the much stronger activity of extracts and fractions of *R. sachalinensis*. 

Meanwhile, according to the literature, the strong antioxidant activity of *R. japonica* rhizomes is often associated with high amounts of stilbenes, mainly resveratrol [6,42,43]. However, there is some evidence that other compounds are co-responsible for high antioxidant activity of rhizomes of *Reynoutria japonica*. As shown by Pan et al. [9], ethanol extract from *Polygon cuspidati rhizoma* was stronger than resveratrol in DPPH and hydroxyl radical scavenging, metal reducing capacity, and preventing of polyunsaturated lipids peroxidation. Also, in the study of Lee et al. [7], no correlation was observed between the content of resveratrol or emodin and antioxidant activity. These results suggest the importance other polyphenols or another group of compounds for determination of antioxidant properties of *R. japonica* rhizomes. Research of Lachowicz et al. [44] indicates a significant influence of procyanidins on antioxidant activity; flavan-3-ols derivatives such as catechins and procyanidins as well trans-piceid and trans-resveratrolside had greater radical scavenging capacity than other compounds observed in *R. japonica* and *R. sachalinensis* extracts.

DPPH scavenging activity and inhibition of lipid peroxidation of proanthocyanidins was investigated in numerous studies [45,46]. Proanthocyanidins are strong DPPH scavengers, e.g., the DPPH IC_50_ values for procyanidin A2 and procyanidin B2 are 2.29 and 3.14 μg/mL, respectively [47]. The scavenging activity of proanthocyanidins increases with the number of hydroxyl groups, especially if they are in the benzene *ortho* position.

Furthermore, polymerization up to trimers increases, but further polymerization decreases the scavenging activity. Higher scavenging activity was found for galloylated procyanidins [45,46]. Among various type of polyphenols, dimeric procyanidins were the most active in scavenging of ABTS and hypochlorous acid and in the FRAP test, followed by flavanols, hydroxycinnamic acids, simple phenolic acids [48]. Taking the above into account it is very likely that procyanidins, including many procyanidins gallate derivatives in the ethyl acetate fractions from the studied *Reynoutria rhizomes* were largely responsible for strong radical scavenging activity. Proanthocyanidins are also good inhibitors of lipid peroxidation, with potency similar or higher than Trolox and vitamin E [45].

Total antioxidant capacity expressed as the ascorbic acid equivalent (AAE) was based on the reduction of Mo(VI) to Mo(V) at acidic pH by the extracts and fractions and the formation of a green phosphate/Mo(V) complex [49]. It appears that unlike ascorbic acid, the compounds (including proanthocyanidins) in the fractions reduced the Mo ions only at a higher (90 °C) temperature (Table 2). The result may be due to the degradation of high polymerized procyanidins at high temperatures and the formation of less polymerized, more active dimer procyanidins. This assumption confirms the study of Luo et al. [50] which developed method for degradation of grape proanthocyanidin polymers into oligomers by sulphurous acid in high temperature (60–80 °C) which resulted in many individual procyanidins dimers and trimers. It was also observed that high polymeric procyanidins exhibited lower values of their half-life times in higher temperature than dimeric procyanidins [51].

Rhizomes of *R. japonica* are known as good source of stilbenes [52,53,54,55] and antraquinones [53,54,55,56]. The European [3] and Chinese Pharmacopeias [57] require determining the content of two compounds, emodin and piceid, in rhizomes of *R. japonica.* However, results of our study suggest that procyanidins should also be considered as compounds affecting the total antioxidant potential of the raw material.

## 3. Materials and Methods

### 3.1. Plant Material

We collected the plant raw material (rhizomes) from a wild population in the disturbed urban areas in the city of Wroclaw (Poland). The rhizomes were harvested in September 2016, when the plants were just before the onset of dormancy. Aerial parts growth and development was completed, leaves still green and fruit abscission beginning. The precise locations of the collection sites are as follows: *R. japonica* (51° 07.404′ N 17° 04.146′ E), *R. sachalinensis* (51° 06.190′ N 17° 08.635′ E), *R.* x *bohemica* (51° 05.666′ N 17° 01.746′ E). Identity of the species was confirmed by Botanical Garden of Medicinal Plants Herbarium staff (Klemens Jakubowski, MSc Botany) based on morphology of the vegetative and generative organs (according to available floras). Voucher specimens were deposited in the Botanical Garden herbarium under deposition numbers AAB1022, AAB1023, AAB1024. The extraction and further sample processing were performed as described previously [5]. In brief, 400 g of air-dried and powdered rhizomes of all three species were extracted four times (2 h each, drug-to-solvent ratio 1:5) with 70% aqueous acetone using an ultrasonic bath (Intersonic IS-36, Olsztyn, Poland). The solvent was evaporated under reduced pressure in a rotary evaporator and 73.75 g, 70.38 g, 79.87 g of *R. japonica*, *R. sachalinensis* and *R.* x *bohemica* acetone dried extracts were obtained, respectively. Fifty g of the raw 70% acetone extract were suspended in water (500 mL) and partitioned between dichloromethane (CH_2_Cl_2_), diethyl ether (Et_2_O), ethyl acetate (AcOEt) and finally butanol (*n*-BuOH) affording 0.97, 1.05, 5.11, 18.91 g of each dried fraction for *R. sachalinensis*, 2.29, 3.09, 6.80, 13.54 g for *R. japonica* and 1.42, 1.835, 8.68, 14.3 g for *R*. x *bohemica* and for the all-water residue fraction. Obtained dried extracts and fractions were weighed accurately, dissolved in 80% MeOH in volumetric flasks to get a 5 mg/mL concentration. Before injection into the HPLC system (Dionex, Idstein, Germany), the solutions were filtered through a 0.22 μm Chromafil syringe polyester membrane (Macherey-Nagel, Düren, Germany) directly to autosampler vials and stored in darkness at 4 °C. The minimum number of replicates for HPLC analyses was three.

### 3.2. Reagents

2,2-Diphenyl-1-picrylhydrazyl (DPPH), thiobarbituric acid (TBA), and hide powder were purchased from Sigma-Aldrich (Steinheim, Germany). Linoleic and gallic acid were purchased from Fluka AG (St. Gallen, Switzerland), and trichloroacetic acid from Ubichem (Redditch, UK). Vanicoside A and vanicoside B were earlier isolated according procedure described in previous article [5]. All other reagents and solvents were obtained from Avantor-POCh, (Gliwice, Poland).

### 3.3. DPPH Scavenging Assay

The ability to scavenge the DPPH free radical was monitored according to a modified method of [58]. Briefly, DPPH solution (0.3 mM) was prepared in methanol. The extract and fractions were dissolved in a mixture of methanol and water (9:1, *v*/*v*) to obtain stock solution (1 mg/mL). Then each stock solution was diluted to obtain final concentrations of 1–250 μg/mL in the assay mixture. DPPH solution (125 μL) and 125 μL of the test extract and fractions at different concentrations were added to a 96- well plate. The absorbance at 517 nm was measured 30 min after mixing using a microplate reader (μQUANT, BioTek, Winooski, VT, USA). The percentage of scavenged DPPH was then calculated according to Equation (1):

%DPPH = ((Abt − Abr)/Ab0) × 100(1)

where Abt is the absorbance of DPPH solution with the test extracts, Ab0 is the absorbance of DPPH solution with a mixture of methanol and water (9:1, *v*/*v*) and Abr is the absorbance of the test extract solution with the addition of methanol. The antiradical activity of extracts was expressed as an EC_50_ value. 

### 3.4. Phosphomolybdenum Reduction Assay

The antioxidant capacity of the extract and fractions was assessed as described by Prieto et al. [49], with modifications. Extract and fractions were dissolved in a mixture of methanol and water (9:1 *v*/*v*) to obtain stock solution (5 mg/mL). Then each stock solution was diluted to obtain final concentrations of 10–500 μg/mL in the assay mixture. The extract and fractions were combined with the reagent solution containing ammonium molybdate (4 mM), sodium phosphate (28 mM) and sulfuric acid (600 mM). The reaction mixture was incubated in a water bath at either 37 °C or 90 °C for 90 min. The absorbance of the colored complex was measured at 695 nm. The antioxidant activity was compared with that of ascorbic acid in the same concentration range and was expressed as the ascorbic acid equivalents (AAE). 

### 3.5. Inhibition of Linoleic Acid Peroxidation

The procedure of Wozniak et al. [59] using Fenton reaction- induced lipid peroxidation, has been adapted for this assay. The extract and fractions dissolved in water, achieved a concentration range of 10–500 μg/mL in the assay mixture. Each fraction (150 μL) was mixed with 500 μL phosphate buffer (0.1 M, pH 7.4), and 550 μL linoleic acid emulsion (linoleic acid mixed with Tween 80, 3:1, *w*/*w*); next 1.12 g emulsion was mixed with 50 mL 0.1 M phosphate buffer (pH 7.4)), and 150 μL 10 mM ascorbic acid. The peroxidation was started with the addition of 150 μL 10 mM FeSO_4_. The reaction mixture was incubated for 90 min. at 37 °C. Thereafter, 1.5 mL of 10% ice cold trichloroacetic acid was added and 1.5 mL of 1% thiobarbituric acid in 50 mM NaOH. The samples were heated in a water bath at 90 °C for 10 min. After cooling the samples, 2 mL of *n*-BuOH was added and mixed well. The absorbance was read at 532 nm after transferring 300 μL of BuOH phase from samples to the 96-well plate. The percentage of linoleic acid peroxidation inhibition was calculated as in [59] using appropriate controls. The inhibition of linoleic acid peroxidation of extracts was expressed as an IC_50_ value.

### 3.6. Total Polyphenols and Tannins Content

Total phenolic content was determined with the Folin-Ciocalteu reagent according to a procedure described previously [60]. Tannin compounds were measured by parallel experiments with extracts vortexed for 1 h with 10 mg/mL hide powder. The results were expressed as gallic acid equivalents according to the standard gallic acid calibration curve. Total tannins were calculated by subtraction of polyphenols non-absorbed by hide powder from the total phenol content. 

### 3.7. HCl-Butanol Assay

Quantification of proanthocyanidins (i.e., procyanidins and delphinidins) in the *Reynoutria* species extracts and fractions was performed in three replicates using the acid butanol method (Bate-Smith method) [40]. (Proanthocyanidins contained in 1 mL of *Reynoutria* species extracts or fractions (at 1 mg/mL) were oxidatively cleaved to anthocyanidins (i.e., cyanidins and delphinidins) at 95 °C for 50 min by adding 6 ml of acid- butanol reagent (butanol/12 N HCl; 95/5; *v/v*) and 200 μL of 2% (*w*/*v*) NH_4_Fe^III^(SO_4_)_2_ × 12 H_2_O (in 2 mol/L HCl). The reaction mixture was cooled and anthocyanidins quantified spectrometrically for absorbance at 550 nm. Blank spectra were obtained for each extract before boiling. 

### 3.8. DMACA-HCl Assay for Flavanols

*Reynoutria* species extracts or fractions (770 μL, at 0.1 mg/mL) were mixed with methanol (385 μL) and DMACA reagent (192 μL), left at room temperature for 20 min and the absorbance at 643 nm was measured [61]. The DMACA reagent was prepared immediately before use, containing 2% (*w*/*v*) DMACA in a cold mixture of methanol and 6 M HCl (1:1, *v*/*v*). 

### 3.9. HPLC-MS Apparatus

For the HPLC analyses, we used Ultimate 3000 series system (Dionex, Idstein, Germany) consisting of dual low-pressure gradient pump with vacuum degasser, an autosampler, a thermostatic column compartment, a diode array detector, an Amazon SL ion trap mass spectrometer with the ESI ion source (Bruker Daltonik, Bremen, Germany) and Corona Ultra RS charged aerosol detector (Thermo Scientific, Bellefonte, PA, USA). 

### 3.10. HPLC-DAD–MS^3^ Conditions

For separation, we used the Kinetex XB C18 150 mm × 2.1 mm × 1.7 μm analytical column (Phenomenex, Torrance, CA, USA), maintained at 25 °C. The following multi-step gradient was used: 0–50 min 15–70% B, 50–55 min 70–95% B, 55–60 min 95% B. The mobile phase “A” (0.1% HCOOH in water), mobile phase “B” (0.1% HCOOH in MeCN), the flow rate was 0.3 mL/min during analysis. 4 μL of each sample was injected to the column by the autosampler. The column was equilibrated for 10 min between injections. UV–vis spectra (Dionex, Idstein, Gemany) were recorded in the range of 200–450 nm. The eluate was introduced into mass spectrometer (Bruker Daltonik, Bremen, Germany) in splitless mode. The parameters for ESI source were: nebulizer pressure 40 psi; dry gas flow 9 L/min; dry temperature 300 °C; and capillary voltage 4.5 kV. Analysis was carried out using scan from *m*/*z* 70 to 2200. Compounds were analyzed in negative ion mode. The parameters for Dissect:Internal S/N threshold −5; Max. number of overlapping compounds-3; Spectrum type-auto; Cut-off intensity −0.1%. The identification of constituents found in plant materials was based on DAD and negative ionization mode MS spectra.

### 3.11. Statistical Analysis

Each of the antioxidant tests and analysis of total polyphenols and tannins was made in three independent experiments, assayed in triplicate. Significant differences (*p* ≤ 0.05) between mean values were evaluated by one-way ANOVA and Duncan’s multiple range test using Statistica 13.1 (Statsoft, Krakow, Poland); results are given in Supplementary Materials. Spearman’s rank order correlation were calculated using Statistica 13.1 Correlation between the peak area of detected compounds (established by using mass spectral deconvolution) and activity of extracts/fractions (1/EC_50_ DPPH, Reducing power AAE 37, 90 (%), 1/EC50 of LA peroxidation) was described with the statistical methods-correlation matrix using Statistica 13.1. Mass spectral deconvolution—the dissect command in Data Analysis TM software (version 4.2, Bruker Daltonics, Billerica, MA, USA) was used to automatically find peak area of compounds on an LC-MS chromatogram trace. The Dissect algorithm utilises fuzzy logic algorithms, which allow a peak separation process to be run without the need for user interaction or any prior information. The parameters for Dissect algorithm: Internal S/N threshold −5; Max. number of overlapping compounds-3; Spectrum type-auto; Cut-off intensity −0.1%. The Principal Component Analysis (PCA) by involved of the LC-MS data (peak area of detected compounds established by using mass spectral deconvolution) and antioxidant assays was performed using Simca-P software (version 15.0.2, Umetrics, Umea, Sweden). Pareto (Par) scaling method with centered and normalized in units of standard deviation were applied to PCA. 

## 4. Conclusions

Fractionation of *Reynoutria* species extracts allowed us to evaluate compounds present in studied raw materials even in small amounts. HPLC/UV/ESI-MS analysis revealed 171 compounds, a total number of 134 constituents were annotated unambiguously (20) or tentatively (114).

Many of identified compounds were observed for the first time in the studied materials. The rhizomes of all species are a rich source of proanthocyanidins. We confirmed the presence of procyanidins with high degree of polymerization, up to decamers, in the rhizomes of *R. japonica* and brought new data on the presence of these compounds in other *Reynoutria* species. A procyanidin trimer digallate was described for the first time in the studied plants. Moreover, we suggest a presence of new, for these species, dianthrone glycosides (emodin bianthrone, emodin bianthrone-hexose, emodin bianthrone-dihexose, emodin bianthrone-hexose-malonic acid, emodin bianthrone-hexose-(malonic acid)-hexose and their methyl or undefined derivatives) that, however, need to be confirmed by isolation and structure elucidation. Fractionation has also allowed to observe the numerous and previously unrecorded phenylpropanoid disaccharide esters (tatariside E, tatariside A, tatariside C, lapathoside B, hydropiperoside B, vanicoside E their isomers and undefined derivatives of phenylpropanoid disaccharide esters) and hydroxycinnamic acid derivatives (dihydroksyferuloyl-*O*-acetoxy-*p*-coumaroyl-*O*-caffeoylquinic acid, (diacetoxymethoxyphenyl) acroyl-*O*-*p*-coumaroyl-*O*-caffeoylquinic acid and its acetyl derivatives), mainly in *R. sachalinensis*. Furthermore, compounds tentatively annotated as lignin oligomers (trimer lignin β-*O*-4-linked S unit with syringaresinol [*S*-(β-*O*-4′)-*S*-(β-β′)-*S*], tetramer lignin, *S*-(8-*O*-4′)-S-(8-*O*-4′)-S-(8-8′)-*S*, derivative of lignin-*S*(8–8)*S*, hedyotisol and its isomers) were observed for the first time in the dichloromethane fractions obtained from the studied species. Other compounds that have been observed for the first time are: *N*-feruloylmethoxytyramine, isovitexin or vitexin diglucoside and slightly suggested: α-carboxyethylhydroxychroman and cyanidin.

The rhizomes of all *Reynoutria* species exhibited strong antioxidant activity. The ethyl acetate fractions, rich in proanthocyanidins, also in galloylated form, were the most active in all antioxidant tests. Statistical analysis demonstrated that proanthocyanidins should be taken considered important contributors to the total antioxidant capacity.

## Figures and Tables

**Table 1:** Retention times, MS data, and UV λ_max_ values of the constituents detected in the extracts and fractions of the three *Reynoutria* species.

Nr.	Identification	Rt	λ max (nm)	*m*/*z* [M − H]^−^	MS^2^ ions	MS^3^ ions	NL Da	References
**1**	Unknown carbohydrate/e.g., Disaccharide-sucrose	1.2	ND	341.15	**178.82b**	160.81b, 142.78	162	[10]
**2**	Unknown carbohydrate	1.21	ND	683.18	341.04b			
**3**	Unknown carbohydrate	1.3	ND	781.12	**439.02b**	420.95, 341.09 [M − 2H]^2−^, 277.01b, 178.80	162	
**4**	Unknown carbohydrate	1.4	ND	781.12	**439.04b**	421.04, 340.98 [M − 2H]^2−^ b, 276.87, 178.83	162	
**5**	Galloyl-glucose	1.5	210, 276	331.13	270.72, 168.58b			[11]
**6**	Unknown	1.8	235, 275, 325	477.1	459.05b, 357.04, 234.83, 150.80			
**7**	Procyanidin dimer, Type B	1.9	225, 280	577.11	559.04, 450.99, **424.96b**, 407.15, 288.93, 286.97	406.90b, 381.02, 272.85	152	[11,12,13]
**8**	Unknown	2.0	235, 275, 325	439.00b, **425.05**	344.98, 240.80b			
**9**	Procyanidin dimer, Type B	2.3	225, 280	577.13	559.04, 450.97, **424.95b**, 407.09, 288.93, 286.97	406.91b, 381.12, 339.07, 272.90	152	[11,12,13]
**10**	Procyanidin trimer, Type B	2.4	225, 280	865.19	739.14, 695.12b, 577.07, 406.98, 286.87			[12,13]
**11**	Catechin *	2.6	225, 280	288.99	270.90, 244.91b, 204.85, 178.83			
**12**	Procyanidin trimer monogallate	2.7	225, 280	1017.2	891.18, 865.18, 847.12, 729.12b, 577.11, 407.07, 287.81			[13]
**13**	Procyanidin dimer, Type B	2.8	225, 280	577.08	559.05, 451.00, **424.96b**, 407.00, 288.90, 286.97	406.90b, 381.11, 272.87	152	[11,12,13]
**14**	Procyanidin pentamer	3.1	225, 280	720.55 [M − 2H]^2−^	1315.33, 1151.29b, 1027.23, 863.22, 635.05, 577.05, 288.85			[12]
**15**	Procyanidin trimer, Type B	3.2	225, 280	865.21	739.13, 695.14b, 577.08, 407.00, 286.90			[12,13]
**16**	Epicatechin *	3.5	225, 280	288.82	270.76, 244.75b, 230.68, 204.70, 178.65			
**17**	Procyanidin dimer monogallate	3.6	225, 280	729.17	**577.06b**, 425.06, 407.07, 286.92	559.05, 450.98, 424.98, 407.00b, 288.90	152	[11,13]
**18**	Procyanidin trimer monogallate	3.7	225, 280	1017.2	**865.16b**, 847.15, 729.11, 577.06, 406.97	847.14, 695.12b, 577.05, 394.95, 286.81	152	[13]
**19**	Procyanidin trimer, Type B	4.0	225, 280	865.2	739.15, 695.14b, 577.07, 406.99, 286.89			[12,13]
**20**	Piceatannol glucoside *	4.1	220, 305, 318	405.06	**242.73b**	224.70b, 214.68, 200.69, 184.64, 174.73	162	
**21**	Procyanidin trimer, Type B	4.2	225, 280	865.19	739.15, 695.12b, 577.08, 406.98, 286.87			[12,13]
**22**	Resveratroloside *	4.6	219, 304, 315	389.07, **435.13** [M + HCOO]^−^	389.07b, 226.91			
**23**	Procyanidin trimer monogallate	4.8	225, 280	1017.19	**865.14**, 847.15, 729.16b, 603.09, 559.08, 407.06, 288.89	847.07b, 695.02, 575.94, 451.02, 286.80	152	[13]
**24**	Procyanidin dimer monogallate	5.1	225, 280	729.12	**577.05**, 559.05, 451.00, 441.01, 407.02b, 288.90	559.01b, 450.99, 406.95, 288.86	152	[11,13]
**25**	Procyanidin tetramer, Type B	5.3	225, 280	1153.26	**1001.20**, 983.20, 865.16b, 739.12, 575.09, 449.02	983.18b, 804.93, 533.18, 382.95	152	[12,13]
**26**	Procyanidin pentamer	6.3	225, 280	720.55 [M − 2H]^2−^	1315.33, 1151.29b, 1027.23, 863.22, 635.05, 577.05, 288.85			[12]
**27**	Piceid *	7.4	218, 308, 318	**389.12**, 435.07 [M + HCOO]^−^	226.71b			
**28**	Procyanidin trimer digallate	7.6	225, 280	1169.24	1151.24, 999.21, 881.22b, 729.18, 603.11, 406.98			[13]
**29**	Procyanidin dimer digallate, Type B	7.7	225, 280	881.16	**729.12b**, 559.08, 407.01, 288.86	603.07, 577.08, 559.07, 451.01b, 407.10, 288.98	152	[13]
**30**	Procyanidin trimer monogallate	7.9	225, 280	1017.22	**865.17**, 847.15, 729.13b, 603.09, 575.09, 406.98, 286.88	847.07b, 739.12, 714.02, 577.04, 448.84, 288.69	152	[13]
**31**	**Procyanidin heptamer**	**8.1**	**225, 280**	**1008.63 [M − 2H]^−^, 1152.74, 1021.26, 999.18, 631.20, 567.10, 499.09b**	**484.00b, 452.98, 419.04, 345.92, 314.85, 288.79**			[12,14]
**32**	Epicatechin-3-*O*-gallate *	8.2	220, 280	440.95	330.82, 302.80, 288.82b, 270.81, 244.82			
**33**	Procyanidin dimer, Type B	8.4	225, 280	577.09	559.08, 450.96, **424.94b**, 407.06, 288.92	406.91b, 381.04, 339.00, 272.85	152	[11,12,13]
**34**	Procyanidin octamer	8.8	225, 280	1152.70 [M − 2H]^−^, 901.21, 879.11, 507.02, **439.04b**	423.93b, 392.86, 358.98, 315.84			[15]
**35**	Procyanidin trimer monogallate	9.0	225, 280	1017.21	**865.12**, 847.15, 729.15b, 603.08, 406.99, 288.90	847.15b, 684.05, 518.85, 451.83, 395.07, 301.69	152	[13]
**36**	Procyanidin octamer	9.1	225, 280	1152.69 [M − 2H]^2−^, 901.16, 864.10, 845.10, 439.02, **382.90b**	302.80b, 284.83, 176.67			[15]
**37**	Procyanidin tetramer monogallate	9.8	225, 280	**652.11** [M − 2H]^2−^ b, 1305.32	1179.22, 1017.23, 863.18, 729.11, 576.00, 567.07b, 440.99, 288.88, 286.86			[15]
**38**	Unknown	10.3	225, 280	1532.47, **382.90b**	302.77b, 284.88, 178.67			
**39**	Procyanidin gallate	10.5	225, 280	**796.27** [M − 3H]^3^ b,948.11 [M − 2H]^2^	1467.40, 1305.34, 1179.31, 1017.21, 863.17, 729.15b, 440.96, 288.86			[15]
**40**	Procyanidin trimer monogallate	11.0	225, 280	1017.2	**891.16**, 847.16, 729.14b, 603.07, 559.05, 407.03, 288.87			[13]
**41**	Procyanidin gallate	11.3	225, 280	**660.32**, 505.17b	1151.19, 999.17, 881.14, 584.04b, 440.96, 302.86			[12,13,15]
**42**	Resveratrol-hexoside	11.4	219, 304, 315	389.06	226.71			
**43**	Procyanidin dimer monogallate	12.0	225, 280	729.13	711.11, 603.05, **577.04**, 559.05, 407.01b, 288.91	559.03, 450.97, 406.93b, 288.85	152	[11,13]
**44**	Emodin glucoside *	12.8	220, 247, 269, 281, 423	431.3	**268.75b**	239.63, 226.68, 224.72b	162	
**45**	Resveratrol *	13.5	218, 306, 318	226.78	184.60, 158.67b, 142.68			
**46**	Procyanidin dimer digallate, Type B	13.6	225, 280	881.13	**729.11b**, 559.12, 407.05, 288.90	603.07, 577.08, 559.04, 451.01b, 407.04, 288.98	152	[13]
**47**	*N*-*trans*-feruloyltyramine *	14.4	220, 281, 323	312.08	296.97b, 177.83, 134.87			
**48**	Acetyl lapathoside d	14.7	220, 290, 315	675.24	633.17, 615.12, **529.07b**, 511.12, 487.12, 453.11, 306.97	487.04b, 469.19, 306.96	146	[16]
**49**	*N*-Feruloyl-methoxytyramine	15.0	220, 281, 323	342.14	327.04b, 308.97, 297.01, 177.84, 134.87			[17]
**50**	Phenylpropanoid-derived disaccharide ester	15.3	220, 284, 315	655.21	613.18b, 595.18, 571.16, 553.10, 425.12, 306.99			
**51**	Cyanidin	15.4	210, 286, 332	286.9	268.79, 150.59b, 134.71, 124.75, 106.72			[18]
**52**	Unknown	15.6	225, 287, 315	585.28	537.17b, 371.13, 359.13			
**53**	**Unknown**	**15.7**	**225, 287, 315**	**583.27**	**535.23b, 369.10, 357.25, 194.91**			
**54**	Unknown	15.8	220, 280	685.23	643.20b, 625.20, 601.19, 337.12, 192.90			
**55**	Unknown	15.9	220, 287, 315	585.29	537.20b, 359.12, 345.14			
**56**	Torachrysone glucoside *	16.2	225, 267, 325	407.16	**244.87b**	229.97	162	
**57**	Unknown	16.8	225, 280	371.12	327.08b, 297.08			
**58**	Unknown	16.85	225, 280	597.26	553.13, 549.23, 383.11b, 371.12, 194.86			
**59**	Unknown	16.9	225, 280	583.31	553.20, 369.11b, 357.15, 194.80			
**60**	Emodin glucoside *	17.0	221, 247, 269, 281, 423	431.04	310.84, 292.76, **268.75b**	264.71, 240.73, 224.70b	162	
**61**	Dihydroksyferuloyl-*O*-acetoxy-*p*-coumaroyl-*O*-caffeoylquinic acid	17.4	214, 282, 325	735.27	693.19, **559.12b**, 541.18	517.11b, 499.10, 337.04, 264.90, 192.83	176	[18]
**62**	Tatariside e	17.5	220, 290, 315	717.39	675.19, **571.11b**, 529.20, 453.12, 288.94	529.06b, 511.05, 469.03, 306.85	146	[19]
**63**	Tatariside e	17.7	220, 290, 315	717.4	675.19, **571.13b**, 529.24, 453.10, 288.93	529.05b, 511.05, 469.00, 306.85	146	[19]
**64**	Unknown	17.8	225, 280	314.95	299.78b, 270.98, 246.72, 204.68, 178.78			
**65**	(diacetoxy-methoxyphenyl)acroyl-*O*-*p*-coumaroyl-*O*-caffeoylquinic acid	17.9	214, 282, 325	777.25	735.22b, 717.25, 693.00, **601.16**, 559.13, 337.09	559.13b, 541.11, 499.05	176	[18]
**66**	Emodin bianthrone-hexose-(malonic acid)-hexose	18.1	220, 278	919.21	875.23, **757.10**, 713.20b, 671.25, 509.08, 458.00	713.15b, 509.04, 502.00, 457.99, 253.79	162	[20,21]
**67**	Derivative of Emodin bianthrone-hexose-malonic acid	18.2	220, 278	1005.23	961.13, 917.29, 757.10, 713.23b, 458.10			[21]
**68**	Unknown	18.3	225, 280, 325	811.36	793.32b, 763.38, 745.34, 669.23, 567.21, 389.09, 342.99, 311.93			
**69**	Unknown	18.4	225, 280, 325	597.27	549.18b, 401.11, 357.12, 342.12, 194.87			
**70**	(diacetoxy-methoxyphenyl)acroyl-*O*-*p*-coumaroyl-*O*-caffeoylquinic acid	18.7	214, 282, 325	777.26	735.24b, 717.25, 693.00, **601.16**, 559.20, 337.04	559.13b, 541.17, 499.13	176	[18]
**71**	Trimer lignin β-*O*-4-linked S unit with syringaresinol [*S*-(β-*O*-4′)-S-(β-β′)-*S*]	19.0	220, 280	643.29	613.22, 417.13b, 387.15, 224.93, 194.87			[22]
**72**	Tetramer lignin, *S*-(8-*O*-4′)-S-(8-*O*-4′)-*S*-(8-8′)-*S*	19.1	220, 280	869.39	851.34b, 821.34, 697.27, 643.22, 595.21, 417.15, 387.15			[22]
**73**	Emodin-*O*-(sulfonyl)-glucoside	19.2	214, 280,	511	430.99, 268.73b, 240.74, 224.96			[11,20]
**74**	Lapathoside c	19.5	220, 290, 315	809.28	**663.13b**, 485.07, 322.98	517.04, 485.10b, 322.88, 280.89	146	[16,23]
**75**	(diacetoxy-methoxyphenyl)acroyl-*O*-*p*-coumaroyl-*O*-caffeoylquinic acid	19.6	214, 282, 325	777.3	735.24b, 717.13, 693.13, **601.17**, 559.00, 337.10	559.13b, 541.13, 499.00	176	[18]
**76**	Lapathoside c isomer	19.7	220, 290, 315	809.28	**663.13b**, 485.07, 322.98	517.04, 485.10b, 322.88, 280.89	146	[16,23]
**77**	Unknown	19.8	220, 280, 315	**327.26**	309.12, 291.10, 228.95b, 210.95, 170.91			
**78**	Emodin-8-*O*-(6’-*O*-malonyl)-glucoside *	19.81	220, 282, 423	517.05	472.99b, 431.10			
**79**	Oligolignol-hedyotisol	20.1	220, 280	809.36	791.33, 773.34, 761.25, 743.33b, 565.21, 417.11			[24]
**80**	Tatariside e	20.2	220, 290, 315	717.22	675.17, **571.09b**, 529.10, 511.17, 487.09	529.05b, 511.04, 487.03	146	[19]
**81**	Derivative of lignin-*S*(8–8)*S*	20.5	220, 280	641.32	623.22, 611.20b, 417.13, 387.08, 347.09, 222.87			[25]
**82**	Unknown	20.7	220, 280, 315	1035.48	1017.45b, 999.38, 969.41, 821.41, 791.35, 595.14			
**83**	Tatariside a	20.8	220, 290, 315	759.22	717.21b, **613.13**, 571.13, 453.04, 288.94	571.09b, 553.10, 529.07, 511.06, 306.71	146	[19]
**84**	Methyl derivative of Emodin bianthrone-hexose-(malonic acid)-hexose	21.0	220, 278	933.21	889.37b, 727.21, 458.06			[21]
**85**	**Oligolignol-e.g., hedyotisol(isomer)**	**21.1**	**220, 280**	**809.37**	**791.34b, 773.25, 761.31, 743.34, 565.21, 417.15**			[24]
**86**	Acetyl derivative of (diacetoxy-methoxyphenyl) acroyl-*O*-*p*-coumaroyl-*O*-caffeoylquinic acid	21.2	214, 282, 325	819.29	777.29b, 759.25, **643.19**, 601.14, 513.13	601.10b, 583.16, 559.07, 337.02	176	[18]
**87**	Hydropiperoside *	21.3	220, 290, 315	779.26	**633.16b**, 615.19, 487.13, 469.16, 453.09	487.12b, 469.16, 453.11, 307.10, 289.03	146	
**88**	(3,6-*O*-di-*p*-coumaroyl)-β-fructofuranosyl-(2→1)-(2′-*O*-acetyl-6′-*O*-feruloyl)-β-glucopyranoside *	21.5	220, 290, 315	851.25	809.23, **705.20b**, 675.20, 527.07	663.22b, 645.38, 559.16, 527.16, 485.12	146	
**89**	Derivative of Emodin bianthrone-di-hexose	21.6	220, 278	1019.22	975.25, 931.42b, 889.25, 727.18, 458.06			[21]
**90**	Acetyl derivative of (diacetoxy-methoxyphenyl) acroyl-*O*-*p*-coumaroyl-*O*-caffeoylquinic acid	21.7	214, 282, 325	819.28	777.25b, 759.38, **643.18**, 601.14, 513.13	601.18b, 583.18, 559.15, 541.11, 337.02	176	[18]
**91**	Unknown	21.9	220, 280, 315	329.27	311.18, 293.12, 228.95b, 210.96, 170.91			
**92**	Emodin bianthrone-hexose-(malonic acid)-hexose	22.0	220, 278	919.2	875.24, **757.09**, 713.20b, 671.13, 509.06, 458.00	713.18b, 508.96, 501.88, 458.03	162	[20,21]
**93**	Oligolignol-e.g.,hedyotisol (isomer)	22.1	220, 280	809.32	791.30, 773.25, 761.28, 743.29, 611.20b, 565.18, 417.19			[24]
**94**	Phenylpropanoid-derived disaccharide ester	22.2	220, 290, 315	987.31	969.39b, 957.50, 851.27, 823.32, 633.18, 453.09			
**95**	Tatariside a (isomer)	22.5	220, 290, 315	759.4	717.22,675.16, **613.14b**, 571.21, 529.18	571.09b, 553.05, 529.06, 511.06	146	[21]
**96**	Emodin bianthrone-hexose-(malonic acid)-hexose	23.0	220, 278	919.21	875.23, **757.10**, 713.22b, 671.25, 509.09, 458.13	713.16b, 509.00, 501.75, 458.20	162	[20,21]
**97**	Unknown	23.01	220, 280, 315	837.37	819.31, 695.25, 640.23b, 579.18, 347.02			
**98**	Acetyl derivative of (diacetoxy-methoxyphenyl) acroyl-*O*-*p*-coumaroyl-*O*-caffeoylquinic acid	23.1	220, 288, 325	819.26	777.28b, 759.38, **643.17**, 601.25, 513.13, 361.01	601.13b, 583.13, 559.11, 336.97	176	[18]
**99**	Acetyl derivative of (diacetoxy-methoxyphenyl) acroyl-*O*-*p*-coumaroyl-*O*-caffeoylquinic acid	23.3	220, 288, 325	819.28	777.27b, 759.25, **643.17**, 601.25, 513.13, 361.04	601.15b, 583.10, 559.11, 336.97	176	[18]
**100**	Isomer of (3,6-*O*-di-*p*-coumaroyl)-β-fructofuranosyl-(2→1)-(2’-*O*-acetyl-6’-*O*-feruloyl)-β-glucopyranoside or tatariside d	23.4	220, 290, 315	851.39	809.24, **705.19b**, 663.27, 527.12	663.20b, 645.25, 559.13, 527.11, 485.10	146	[19]
**101**	Hydropiperoside isomer	23.45	220, 290, 315	779.36	**633.11b**, 615.25, 487.06, 469.13, 453.38, 288.86	487.06b, 469.18, 453.08, 306.90, 288.88	146	
**102**	Methyl derivative of Emodin bianthrone-hexose-(malonic acid)-hexose	23.5	220, 278	933.21	889.47b, 727.24, 458.09			[21]
**103**	Vanicoside C *	23.8	220, 290, 315	821.23	761.18, **675.16b**, 633.19, 529.10, 487.09, 288.87	633.15, 529.10b, 453.18, 288.98	146	
**104**	Acetyl derivative of (diacetoxy-methoxyphenyl)acroyl-*O*-*p*-coumaroyl-*O*-caffeoylquinic acid	24.0	220, 290, 315	819.31	777.29b, 759.25, **643.17**, 601.25, 583.20, 361.04	601.15b, 583.10, 559.11, 337.13	176	[18]
**105**	Derivative of Emodin bianthrone-hexose-malonic acid	24.1	220, 278	1005.22	961.13, 917.29, 757.12, 713.23b, 458.07			[21]
**106**	Phenylpropanoid-derived disaccharide esters	24.2	220, 290, 315	1181.4	1133.38, 1009.38, 955.50b, 809.41, 663.14			
**107**	Phenylpropanoid-derived disaccharide esters	24.5	220, 290, 315	1151.38	1133.42, 1103.35, 1009.32, 955.40b, 809.29			[23]
**108**	Phenylpropanoid-derived disaccharide esters	24.6	220, 290, 315	1151.4	1133.38, 1103.38, 1009.33, 955.39b, 809.29			[23]
**109**	Unknown	24.7	220, 280, 315	623.28	591.21, 551.26, 486.13, 460.17b, 352.16, 297.07			
**110**	Tatariside b *	25.0	220, 290, 315	893.27	851.24, **747.22b**, 705.27, 687.33, 569.19	705.24b, 687.25, 663.22, 569.16, 527.18, 322.96	146	
**111**	Methyl derivative of Emodin bianthrone-hexose-(malonic acid)-hexose	25.1	220, 278	933.2	889.42b, 727.19, 685.20, 416.06			[21]
**112**	Derivative of Emodin bianthrone-di-hexose	25.4	220, 278	1019.24	975.25, 931.43b, 889.25, 727.20, 458.07			[21]
**113**	Vanicoside B (isomer)	25.6	220, 290, 315	955.37	**809.26b**, 663.19	663.26b, 485.20, 453.09	146	
**114**	**Unknown**	**25.8**	**220, 280, 315**	**801.29**	**759.25b, 741.50, 655.19, 613.25, 571.13, 331.05**	**613.18b, 595.13, 571.15, 553.12, 330.95**	**146**	
**115**	Tatariside b (isomer)	26.0	220, 290, 315	893.28	851.27, **747.21b**, 705.29, 687.31, 569.18	705.26b, 687.37, 663.34, 569.23, 527.31, 322.98	146	
**116**	Vanicoside B *	26.4	220, 290, 315	955.29	**809.22b**, 663.20, 453.05	663.21b, 485.20, 323.05	146	
**117**	Lapathoside a	26.6	220, 290, 315	985.3	839.24b, **809.24**, 663.22, 483.12	663.20b, 485.08, 322.85	176	[16,23]
**118**	Diacetyl derivative of (diacetoxy-methoxyphenyl)acroyl-*O*-*p*-coumaroyl-*O*-caffeoylquinic acid	26.7	220, 288, 325	861.3	819.29b, 801.25, 777.25, 759.25, **685.20**, 643.17, 601.20, 583.18, 559.25, 513.17, 361.01	643.19b, 625.18, 601.15, 583.15	176	[18]
**119**	Lapathoside b	26.8	220, 290, 315	1015.31	869.23, **839.23b**, 693.19, 663.22, 483.15	693.23, 663.20b, 645.28, 499.09, 322.89	176	[26]
**120**	Questin *	27.0	222, 286, 430	282.94	267.89, 239.85b			
**121**	Phenylpropanoid-derived disaccharide esters	27.1	220, 290, 315	1193.48	1175.45, 1145.50, 1051.38, 997.44b, 851.31, 821.30			
**122**	Phenylpropanoid-derived disaccharide esters	27.2	220, 290, 315	1163.41	1145.45b, 1133.51, 999.37, 955.30, 851.15, 809.28			
**123**	Diacetyl derivative of (diacetoxy-methoxyphenyl) acroyl-*O*-*p*-coumaroyl-*O*-caffeoylquinic acid	27.3	220, 288, 325	861.32	819.29b, 801.25, 777.25, 759.25, **685.20**, 643.17, 601.20, 583.18, 559.25, 513.17, 361.01	643.17b, 625.18, 601.15, 583.15	176	[18]
**124**	Vanicoside B (isomer)	27.4	220, 290, 315	955.28	**809.20b**, 663.19, 453.04	663.23b, 485.20, 323.06	146	
**125**	Dihydroferuloyl vanicoside B	27.8	220, 290, 315	1133.4	1115.49b, 1103.65, 997.32, 969.37			[16,23]
**126**	Unknown	28.0	220, 290, 315	1071.38	1053.46b, 1041.64, 935.32, 907.40, 866.38, 717.11			
**127**	Diacetyl derivative of (diacetoxy-methoxyphenyl)acroyl-*O*-*p*-coumaroyl-*O*-caffeoylquinic acid	28.1	220, 288, 325	861.32	819.29b, 801.25, 777.25, 759.25, **685.20**, 643.17, 601.20, 583.18, 559.25, 513.17, 361.01	643.17b, 625.18, 601.15, 583.15	176	[18]
**128**	Emodin bianthrone-hexose-malonic acid	28.2	220, 278, 350	757.14	713.25b, 509.10, 458.12			[21]
**129**	Dihydroferuloyl vanicoside B	28.5	220, 290, 315	1133.38	1115.49b, 1103.50, 997.33, 969.38			[16,23]
**130**	Derivative of Emodin bianthrone-di-hexose	28.7	220, 278	1019.24	975.38, 931.43b, 889.25, 727.20, 458.07			[21]
**131**	Vanicoside A *	29.0	220, 290, 315	997.31	955.29, **851.24b**, 821.28, 705.21, 453.05	809.24, 705.29b, 663.48, 527.22	146	
**132**	Tatariside C	29.1	220, 290, 315	935.27	893.27, **789.22b**, 747.32, 705.29, 611.17, 569.18	747.26b, 705.23, 611.26, 569.22	146	[19,27]
**133**	Hydropiperoside b	29.2	220, 290, 315	1027.3	985.38, 967.30, 881.25b, **851.23**, 705.20, 453.09	809.19, 705.20b, 663.20, 527.08, 453.06, 322.96	176	[28]
**134**	Derivative of (diacetoxy-methoxyphenyl)acroyl-*O*-*p*-coumaroyl-*O*-caffeoylquinic acid	29.4	220, 285, 325	965.36	923.31, **819.26**, 789.29b, 747.22, 643.21	777.31b, 643.08, 611.15, 569.05, 361.06	146	[18]
**135**	Derivative of (diacetoxy-methoxyphenyl) acroyl-*O*-*p*-coumaroyl-*O*-caffeoylquinic acid	29.7	220, 285, 325	995.37	953.33, **819.23b**, 777.25, 759.13, 611.24	777.23b, 735.18, 643.29, 611.16, 569.18	176	[18]
**136**	Isomer vanicoside A/vanicoside F	29.9	220, 290, 315	997.32	955.29, **851.24b**, 821.28, 705.21, 453.06	809.22, 705.27b, 663.31, 527.20, 323.01	146	
**137**	Phenylpropanoid-derived disaccharide esters	30.3	220, 290, 315	1175.43	1157.52b, 1145.61, 1039.33, 1011.37			
**138**	Emodin bianthrone-hexose	30.35	220, 278, 350	671.17	653.18, **509.09**, 416.08b, 253.95	491.01, 253.88b	162	[21]
**139**	Unknown	30.4	220, 265, 325	**324.99b**, 244.93	244.88			
**140**	Unknown	30.7	220, 265, 325	1113.43	1095.45b, 1083.45, 977.29, 949.33			
**141**	Phenylpropanoid-derived disaccharide esters	31.4	220, 290, 315	954.33 [M − 3H]^3^	881.20 [M − 2H]^2^, 809.20, **779.22b**	633.09b, 486.99	176	[23]
**142**	Emodin bianthrone-hexose-malonic acid	31.5	220, 278, 350	757.16	713.25b, 671.25, 509.10, 502.00, 458.12			[21]
**143**	Vanicoside E	32.1	220, 290, 315	1039.31	997.24, **893.25b**, 747.30, 453.05	851.27, 747.28b, 705.40, 569.24, 304.91	146	[27,28]
**144**	Emodin bianthrone-hexose-malonic acid	32.7	220, 278, 350	757.16	713.21b, 671.19, 509.11, 502.00, 458.12			[21]
**145**	Methyl derivative of Emodin bianthrone-hexose	33.0	220, 278, 350	685.18	416.07b, 253.92			[21]
**146**	Methyl derivative of Emodin bianthrone-hexose	34.0	220, 278, 350	685.17	416.07b, 253.92			[21]
**147**	Emodin *	**34.2**	**220, 248, 265, 288, 430**	**268.89**	**240.81, 224.93b, 181.68**			
**148**	Methyl derivative of Emodin bianthrone-hexose-malonic acid	34.6	220, 278, 350	771.14	727.22b, 502.05, 458.07			[21]
**149**	Unknown	35.0	220, 278, 350	721.41	675.39b, 397.10			
**150**	Methyl derivative of Emodin bianthrone-hexose-malonic acid	35.2	220, 278, 350	771.15	727.24b, 502.05, 458.07			[21]
**151**	Methyl derivative of Emodin bianthrone-hexose-malonic acid	35.6	220, 278, 350	771.14	727.23b, 502.04, 458.08			[21]
**152**	Methyl derivative of Emodin bianthrone-hexose-malonic acid	36.3	220, 278, 350	771.15	727.23b, 502.04, 458.08			[21]
**153**	Unknown	37.5	225, 280, 325	**647.37b**, 1203.74	601.34b, 341.1			
**154**	Unknown	37.9	225, 280, 325	723.42	677.40, 397.09			
**155**	Unknown	38.3	220, 278, 350	369.18	351.12, 311.02, 292.99b, 210.79, 170.76			
**156**	Unknown	38.4	225, 280, 325	559.35	513.28b, 277.15, 252.98			
**157**	Unknown	39.4	225, 280, 325	559.36	513.29b, 277.16, 253.01			
**158**	Unknown	40.7	225, 275	649.39	603.37			
**159**	Isovitexin/vitexin diglucoside	41.0	269, 333	755.39	**593.25**, 575.29b, 477.06, 431.21	533.25, 503.21, 431.19b, 413.28	162	[29,30]
**160**	Unknown	41.5	220, 278, 360	725.45	679.43b, 397.09			
**161**	Emodin bianthrone	41.6	220, 278, 360	509.14	491.08, 253.88b			[21]
**162**	Unknown	42.1	225, 280, 325	295.19	277.08b, 194.94, 170.90			
**163**	Unknown	42.7	225, 280, 325	561.59	515.32b, 279.20, 253.00			
**164**	Unknown	42.8	225, 280, 325	625.39	579.36			
**165**	Emodin bianthrone isomer	43.6	220, 278, 360	509.14	491.06, 253.88b			[21]
**166**	Unknown	44.7	225, 280, 325	651.41	605.4			
**167**	Unknown	45.2	220, 278, 350	757.4	**595.30**, 577.30, 477.05b, 433.22, 279.16	535.27, 505.24, 475.23, 433.22b, 279.13	162	
**168**	Unknown	47.2	225, 280, 325	563.39	517.34b, 281.21, 253.00			
**169**	Methyl derivative of emodin bianthrone	50.4	220, 278, 360	523.18	253.89			[21]
**170**	Alpha-carboxyethylhydroxychroman	54.4	292	277.19	259.13, 233.06b			[31]
**171**	Unknown	57.4	220, 278, 350	279.2	261.11b, 233.17			

b-base peak (the most abundant ion in the recorded spectrum), **in bold**—ions subjected to MS/MS fragmentation (if it’s not obvious), *-isolated and/or characterised in our previous paper [5], ND-not determined.

**Table 2:** Antioxidant activity of the studied extracts and fractions.

Fraction	Radical Scavenging Activity DPPH (EC_50_ µg/mL)			Reducing Power AAE (%) 37 °C			Reducing Power AAE (%) 90 °C			LA-Peroxidation (IC_50_ µg/mL)		
	*R.j*	*R.s*	*R.b*	*R.j*	*R.s*	*R.b*	*R.j*	*R.s*	*R.b*	*R.j*	*R.s*	*R.b*
Acetone	9.6 ± 0.5	8.7 ± 0.4	12.6 ± 0.7	6.5 ± 0.3	6.0 ± 0.3	6.4 ± 0.2	28.5 ± 1.1	27.9 ± 1.0	21.4 ± 1.6	80.3 ± 2.8	71.6 ± 2.6	68.9 ± 1.6
Dichloromethane	202.1 ± 5.6	56.5 ± 3.9	63.3 ± 2.9	2.6 ± 0.2	1.8 ± 0.1	1.6 ± 0.06	11.2 ± 0.1	12.2 ± 0.6	10.8 ± 0.4	401.8 ± 12.7	112.2 ± 2.5	153.6 ± 6.0
Diethyl ether	9.3 ± 0.4	10.2 ± 0.8	8.8 ± 0.3	10.2 ± 0.5	8.3 ± 0.4	10.9 ± 0.4	35.0 ± 1.6	32.6 ± 1.2	35.4 ± 1.1	63.8 ± 2.6	67.3 ± 1.4	52.1 ± 2.6
Ethyl acetate	6.5 ± 0.4	4.7 ± 0.3	6.2 ± 0.1	13.9 ± 0.3	16.2 ± 0.2	16.6 ± 0.2	38.8 ± 1.3	44.7 ± 1.3	36.5 ± 1.7	45.7 ± 1.9	32.3 ± 1.7	40.6 ± 1.4
Butanol	9.1 ± 0.3	6.9 ± 0.2	8.1 ± 0.3	6.6 ± 0.2	8.2 ± 0.2	8.1 ± 0.2	29.0 ± 1.1	29.4 ± 0.8	25.7 ± 1.2	93.2 ± 3.5	66.2 ± 2.6	113.4 ± 4.2
Water	58.0 ± 2.5	35.0 ± 0.5	57.3 ± 2.3	0.6 ± 0.02	1.5 ± 0.05	0.1 ± 0.01	13.6 ± 0.4	16.9 ± 0.4	12.8 ± 0.2	650.7 ± 10.6	635.6 ± 17.8	690.1 ± 9.0

Radical Scavenging activity DPPH for ascorbic acid (as control) EC50 = 8.6 ± 0.4 µg/mL; Reducing power AAE (%) for quercetin (as control) at 37 °C = 30.7 ± 1.2 AAE (%) and at 90 °C = 52.0 ± 2.7 AAE (%), LA-Peroxidation for quercetin (as control) IC50 = 19.6 ± 1.1 µg/mL. *R.j*-*Reynoutria japonica*, *R.s*-*Reynoutria sachalinensis*, *R.b*-*Reynoutria* x *bohemica*. Data were expressed as mean ± SD, performed in at least three independent experiments, assayed in triplicate.

**Table 3:** Total polyphenols and tannins content in studied extracts and fractions. Data were expressed as mean ± SD, performed in at least three independent experiments, assayed in triplicate.

Fraction	TPC Total Polyphenols [GAE] mg/g Fraction			Tannins Content [GAE] mg/g Fraction		
	*R.j*	*R.s*	*R.b*	*R.j*	*R.s*	*R.b*
Acetone	324.1 ± 9.8	317.7 ± 14.1	487.7 ± 11.9	233.3 ± 6.4	264.0 ± 7.0	360.0 ± 6.5
Dichloromethane	96.4 ± 5.6	22.7 ± 0.9	81.1 ± 2.7	61.0 ± 2.9	13.0 ± 0.4	60.3 ± 2.7
Diethyl ether	469.1 ± 3.0	355.1 ± 17.1	615.4 ± 6.7	338.6 ± 17.2	241.6 ± 11.3	509.3 ± 19.8
Ethyl acetate	583.4 ± 6.5	640.7 ± 11.0	642.9 ± 8.9	484.3 ± 19.1	528.3 ± 16.9	510.5 ± 15.8
Butanol	307.1 ± 6.9	352.7 ± 7.0	286.1 ±6.0	258.0 ± 9.6	315.0 ± 7.4	243.0 ± 10.4
Water	28.7 ± 1.5	65.4 ± 4.5	29.7 ± 2.2	23.6 ± 1.1	46.6 ± 2.0	29.3 ± 0.6

**Table 4:** Spearman Rank Order Correlation. Marked correlations are significant at *p* < 0.05.

Variable	LA-Peroxidation EC_50_	DPPH EC_50_	Reducing Power AAE 37 °C	Reducing Power AAE 90 °C	Total Polyphenols	Tannins	DMACA	HCl-Butanol
LA-Peroxidation EC_50_	1000	0.751	−0.904	−0.874	−0.823	−0.804	−0.938	−0.300
DPPH EC50	0.751	1000	−0.843	−0.869	−0.663	−0.742	−0.757	−0.736
Reducing power AAE 37 °C	−0.904	−0.843	1000	0.899	0.781	0.819	0.877	0.400
Reducing power AAE 90 °C	−0.874	−0.869	0.899	1000	0.795	0.810	0.917	0.411
Total polyphenols	−0.823	−0.663	0.781	0.795	1000	0.939	0.779	0.259
Tannins	−0.804	−0.742	0.819	0.810	0.939	1000	0.738	0.378
DMACA	−0.938	−0.757	0.877	0.917	0.779	0.738	1000	0.272
HCL-Butanol	−0.300	−0.736	0.400	0.411	0.259	0.378	0.272	1000

**Table 5:** Correlation between the peak area of detected compounds (established by using mass spectral deconvolution) and activity of extracts/fractions (1/EC_50_ DPPH, Reducing power AAE 37, 90 (%), 1/EC_50_ of LA peroxidation) was described with the statistical methods-correlation matrix. In the table are presents only peaks with positive correlation, significant at *p* < 0.05.

Nr.	Identification	EC50linoleic	EC50 DPPH	AAE 37	AAE 90
**9**	Procyanidin dimer	0.563	0.552	0.458	0.484
**10**	Procyanidin trimer	0.63	0.68	0.62	0.572
**11**	Catechin	0.611	0.305	0.373	0.502
**12**	Procyanidin trimer monogallate	0.635	0.646	0.665	0.601
**13**	Procyanidin dimer	0.554	0.536	0.664	0.645
**15**	Procyanidin trimer	0.555	0.571	0.536	0.527
**17**	Procyanidin dimer monogallate	0.763	0.655	0.762	0.795
**18**	Procyanidin trimer monogallate	0.494	0.504	0.446	0.445
**20**	Piceatannol glucoside	0.432	0.389	0.588	0.446
**21**	Procyanidin trimer	0.48	0.512	0.6	0.501
**22**	Resveratrolside	0.342	0.353	0.499	0.491
**23**	Procyanidin trimer monogallate	0.781	0.697	0.806	0.783
**24**	Procyanidin dimer monogallate	0.687	0.684	0.758	0.734
**25**	Procyanidin tetramer	0.481	0.526	0.608	0.518
**26**	Procyanidin pentamer	0.35	0.438	0.584	0.387
**27**	Piceid	0.34	0.319	0.48	0.466
**28**	Procyanidin trimer digallate	0.592	0.598	0.717	0.585
**29**	Procyanidin dimer digallate	0.477	0.414	0.592	0.583
**30**	Procyanidin trimer monogallate	0.494	0.504	0.446	0.445
**35**	Procyanidin trimer monogallate	0.746	0.719	0.764	0.721
**37**	Procyanidin tetramer monogallate	0.682	0.701	0.729	0.643
**39**	Procyanidin gallate	0.666	0.669	0.724	0.618
**40**	Procyanidin trimer monogallate	0.716	0.561	0.753	0.636
**78**	Emodin-8-*O*-(6’-*O*-malonyl)-glucoside	0.37	0.349	0.496	0.316
**87**	Hydropiperoside	0.541	0.212	0.264	0.395
**106**	Phenylpropanoid-derived disaccharide esters	0.659	0.391	0.424	0.509
**107**	Phenylpropanoid-derived disaccharide esters	0.511	0.366	0.458	0.561
**108**	Phenylpropanoid-derived disaccharide esters	0.704	0.477	0.631	0.719
**113**	Vanicoside B (isomer)	0.501	0.166	0.198	0.338
**116**	Vanicoside B	0.618	0.315	0.349	0.473
**117**	Lapathoside a	0.537	0.209	0.263	0.394
**121**	Phenylpropanoid-derived disaccharide esters	0.579	0.41	0.407	0.511
**122**	Phenylpropanoid-derived disaccharide esters	0.556	0.289	0.358	0.447
**124**	Vanicoside B (isomer)	0.564	0.217	0.284	0.403
**125**	Dihydroferuloylvanicoside B	0.624	0.341	0.39	0.54
**141**	Phenylpropanoid-derived disaccharide esters	0.494	0.504	0.446	0.445

## Supplementary Materials

The following are available online at https://www.mdpi.com/1420-3049/24/6/1136/s1, Table S1: Multi-component one-way ANOVA results for Table 2. Table S2: Multi-component one-way ANOVA results for Table 3, Figure 8 and Figure 9.
