# Supplementary material for: Phytochemical Diversity in Rhizomes of Three Reynoutria Species and their Antioxidant Activity Correlations Elucidated by LC-ESI-MS/MS Analysis
Source: Molecules. 2019 Mar 21;24(6):1136. doi: 10.3390/molecules24061136 (PMC6470775; doi:10.3390/molecules24061136)
Supplement: Supplementary file 1 [file molecules-24-01136-s001.pdf]

Table S1. Multi-component one-way ANOVA results for Table 2.

Extract/Fraction	1	2	3	4	5	6	7	8	9	10	11	12	13	14	15	16	17	18
1.Acetone *R.j*		*			**/***	*	***/****	*		*	***	*	**/****	*				*
2.Dichloromethane *R.j*	*		*	*	*	*	*	*/***	*	*	*	*	*	*/***	*	*	*	*/***
3.Diethyl ether *R.j*		*				*	****	*	****	*	****	*	****	*	***	***		*
4.Ethyl acetate *R.j*		*				*		*	****			*	*	*	****	****		*
5.Butanol *R.j*	**/***	*				*	***	*		*	***	*	**	*				*
6.Water *R.j*	*	*	*	*	*		*	***	*	*	*	*	*	*	*	*	*	***
7.Acetone *R.s*	***/****	*	****		***	*		*		*	***/****	*	*/**/****	*				*
8.Dichloromethane *R.s*	*	*/***	*	*	*	***	*		*	*	*	*/**	*	*/**/***	*	*	*/****	***
9.Diethyl ether *R.s*		*	****			*	****	*		*	**/****	*	****	*		*/****	**	*
10.Ethyl acetate *R.s*	*	*	*		*	*	*	*	*			*	*	*	*	**/****		*
11.Butanol *R.s*	***	*	****		***	*	***/****	*	**			*	*/****	*			**	*
12.Water *R.s*	*	*	*	*	*	*	*	*/**	*	*	*		*	*/**	*	*	*	*
13.Acetone *R.b*	**/****	*	****	*	**	*	*/**/****	*		*	*/****	*		*	*	*	*	*
14.Dichloromethane *R.b*	*	*/***	*	*	*	*	*	*/**/***	*	*	*	*/**	*		*	*	*	*
15.Diethyl ether *R.b*		*	***	****		*		*		*		*	*	*		***/****		*
16.Ethyl acetate *R.b*		*	***	****		*		*	*/****	**/****		*	*	*	***/****			*
17.Butanol *R.b*		*				*		*/****			**	*	*	*				*
18.Water *R.b*	*	*/***	*	*	*	***	*	***	*	*	*	*	*	*	*	*	*	

Values that are significantly different at P≤0.05 (* for DPPH), and that are not significantly different at P≤0.05 (** for AAE 37 °C, *** for AAE 90 °C, **** for LA-Peroxidation) determined by ANOVA with Duncan's post-hoc test.

Table S2. Multi-component one-way ANOVA results for Table 3, Figure 8 and Figure 9.

Extract/Fraction	1	2	3	4	5	6	7	8	9	10	11	12	13	14	15	16	17	18
1.Acetone *R.j*							*		**		***		****				**	
2.Dichloromethane *R.j*														*/**/***				***
3.Diethyl ether *R.j*													*	****	***	***		
4.Ethyl acetate *R.j*					****		****									****		
5.Butanol *R.j*	*			****			*/**/****						***			*/**/****	***	
6.Water *R.j*								*				**/***		***				*/**/****
7.Acetone *R.s*	*/***			****	*/**/****						***					**		
8.Dichloromethane *R.s*						*						***						*
9.Diethyl ether *R.s*	**										*				****		**	
10.Ethyl acetate *R.s*											****				*/**			
11.Butanol *R.s*	***						***		*	****								
12.Water *R.s*						**/***		***						*				
13.Acetone *R.b*	****		*		***												***	
14.Dichloromethane *R.b*		*/**/***	****			***						*			****			***
15.Diethyl ether *R.b*			***						****	*/**				****		***		
16.Ethyl acetate *R.b*			***	****	*/**/****		**								***		*	
17.Butanol *R.b*	**				***				**				***			*		
18.Water *R.b*		***				*/**/****		*						***				

Values that are not significantly different at P≤0.05 (* for TPC Total polyphenols, ** for Tannins, *** for DMACA, **** for HCL-Butanol) determined by ANOVA with Duncan's post-hoc test.
